# Forkhead box O proteins: steering the course of stem cell fate

**DOI:** 10.1186/s13619-024-00190-1

**Published:** 2024-03-11

**Authors:** Mengdi Cheng, Yujie Nie, Min Song, Fulin Chen, Yuan Yu

**Affiliations:** 1 Laboratory of Tissue Engineering, College of Life Sciences, Northwest University, Xi’an, China; 2 Provincial Key Laboratory of Biotechnology of Shaanxi, Northwest University, Xi’an, China; 3 Key Laboratory of Resource Biology and Biotechnology in Western China, Ministry of Education, School of Medicine, Northwest University, Xi’an, China

**Keywords:** Stem cell fate, Self-renewal, Quiescence, Differentiation, FOXO

## Abstract

Stem cells are pivotal players in the intricate dance of embryonic development, tissue maintenance, and regeneration. Their behavior is delicately balanced between maintaining their pluripotency and differentiating as needed. Disruptions in this balance can lead to a spectrum of diseases, underscoring the importance of unraveling the complex molecular mechanisms that govern stem cell fate. Forkhead box O (FOXO) proteins, a family of transcription factors, are at the heart of this intricate regulation, influencing a myriad of cellular processes such as survival, metabolism, and DNA repair. Their multifaceted role in steering the destiny of stem cells is evident, as they wield influence over self-renewal, quiescence, and lineage-specific differentiation in both embryonic and adult stem cells. This review delves into the structural and regulatory intricacies of FOXO transcription factors, shedding light on their pivotal roles in shaping the fate of stem cells. By providing insights into the specific functions of FOXO in determining stem cell fate, this review aims to pave the way for targeted interventions that could modulate stem cell behavior and potentially revolutionize the treatment and prevention of diseases.

## Background

Stem cells represent indispensable entities in the intricate processes of embryonic development, tissue homeostasis, and regeneration, in which the behavior of stem cells is dynamically regulated, leading them to either maintain pluripotency or differentiate in a context-dependent manner (Zhang and Wang 2008). At the core of this process lie the molecular mechanisms that integrate intrinsic and extrinsic factors to orchestrate the ultimate fate determination of stem cells. Dysregulation of the decision-making mechanisms can give rise to diseases such as cancer, neurodegenerative disorders, and tissue degeneration (Chen et al. 2022; Hu et al. 2019; Lu et al. 2019). Therefore, comprehending the intricate mechanisms controlling stem cell fate decisions is essential for unraveling the complexities of development, tissue homeostasis, and regenerative potential.

FOXO proteins, also known as Forkhead box O proteins, are a group of transcription factors involved in a variety of cellular functions, such as cell survival, metabolism, cell cycle regulation, and DNA repair (Huang and Tindall 2007; Rodriguez-Colman et al. 2023; Webb and Brunet 2014). They have been found to play a multifaceted role in stem cell fate determination. For example, in embryonic stem cells (ESCs), FOXOs help to maintain self-renewal by activating the expression of key pluripotency factors like OCT4 and SOX2 (Zhang et al. 2011b). FOXOs also promote the entry of adult stem cells (ASCs) into a quiescent state, helping to preserve the stem cell pool and prevent premature depletion (de Morree and Rando 2023; Gopinath et al. 2014; Paik et al. 2009). Additionally, FOXOs are involved in regulating the differentiation of stem cells into specific lineages. Depending on the context, FOXOs can either promote or inhibit stem cell differentiation (Dengler et al. 2008; Hribal et al. 2003; Kim et al. 2015). The current review delves into the structure and regulation of FOXO transcription factors, with a specific focus on their pivotal roles in steering the decision of stem cell fate. The goal is to provide insights into the specific functions of FOXOs in shaping stem cell fate, potentially leading to targeted interventions to modify stem cell behavior and treat or prevent diseases.

## Structure of FOXO proteins

Since the discovery of the *Drosophila* forkhead (*fkh*) gene in 1989 (Weigel et al. 1989), an increasing number of FOXO genes have been identified in different species. FOXO genes were initially discovered at the sites of chromosomal translocations that occur in human rhabdomyosarcomas and acute myeloid leukemias (Anderson et al. 1998; Borkhardt et al. 1997). Shortly after, the DAF-16 protein, the nematode ortholog of FOXOs, was identified in *Caenorhabditis elegans*. Invertebrates usually possess a single copy of FOXO gene (Bridge et al. 2010; Kenyon et al. 1993; Pascual-Carreras et al. 2021; Puig et al. 2003), whereas mammals have four FOXO members, namely FOXO1 (also known as Forkhead in rhabdomyosarcoma, FKHR), FOXO3 (also know as FKHR like 1, FKHRL1), FOXO4 (also known as acute-lymphocytic-leukaemia-1 fused gene from chromosome X, AFX), and FOXO6 (Orea-Soufi et al. 2022).

**Fig. 1:**
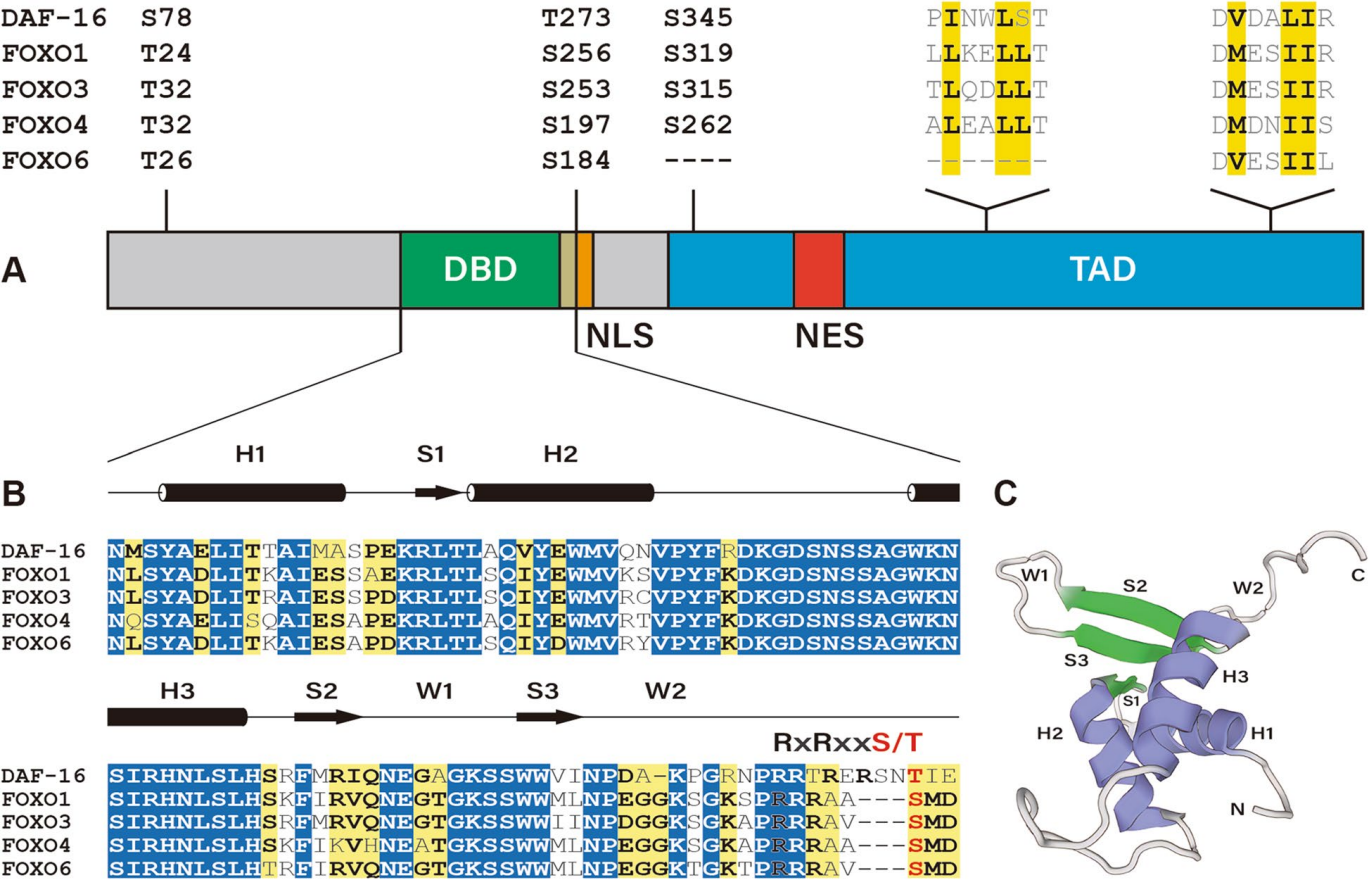
Domain architecture and sequence alignment of FOXO proteins. **A** Schematic domain structure of FOXO proteins. The Forkhead DNA-binding domain (DBD), nuclear localization signal (NLS), nuclear export signal (NES), and transcriptional activation domain (TAD) are colored green, orange, red, and blue, respectively. Conserved AKT phosphorylation sites in mammalian and *C. elegans* FOXO proteins are indicated with corresponding amino acid positions (S for serine, T for threonine). The hydrophobic residues of the “ΦxxΦΦ” motif are shaded in yellow. **B** The sequence alignment of the Forkhead DBDs from FOXO proteins is presented with reference to the secondary structural elements (H for α-helix, S for β-strand, W for wing). Identical and highly conserved amino acids are shaded in blue and yellow, respectively. **C** A three-dimensional structure of the Forkhead DBD is depicted

FOXO proteins consist of four main functional structural domains, including the Forkhead DNA-binding domain (DBD), nuclear localization signal (NLS), nuclear export signal (NES), and transcriptional activation domain (TAD). The Forkhead DBD, spanning about 110 amino acids in length, comprises three α-helices, three β-folds, and two winged loops (Brown and Webb 2018) (Fig. 1A). The highly conserved α-helices are primarily responsible for the interaction of FOXO with DNA through hydrogen bonds and van der Waals forces (Obsil and Obsilova 2011; Tsai et al. 2007) (Fig. 1B and C). While FOXO proteins recognize two distinct response elements, the insulin-responsive element (IRE) and the DAF-16 family member binding element (DBE), they bind to the DBE with a higher affinity (Furuyama et al. 2000; Obsil and Obsilova 2008; Weigelt et al. 2001).

The NLS and NES domains control the subcellular distribution of FOXO proteins by interaction with specific nuclear import and export receptor proteins (Van Der Heide et al. 2004). The NLS overlaps with the C-terminus of FOXO DBD and shares arginine residues with an RxRxxS/T motif (R for arginine, S for serine, T for threonine, and x for any amino acid), in which the serine/threonine residue is phosphorylated by AKT kinase (Brownawell et al. 2001; Zhang et al. 2002). AKT phosphorylates FOXO1, FOXO3, and FOXO4 at three conserved sites, such as Thr32, Ser253, and Ser315 in FOXO3 (Biggs et al. 1999; Takaishi et al. 1999), while FOXO6 only has two of these sites (Thr26 and Ser184) (Jacobs et al. 2003) (Fig. 1A). AKT-mediated phosphorylation creates binding sites for the 14-3-3 protein, leading to the translocation of the resulting FOXO complex to the cytosol, where the bound 14-3-3 protein hinders FOXO’s ability to re-enter the nucleus by potentially masking the NLS (Brunet et al. 2002; Cahill et al. 2001). It should be noted that efficient nuclear export of FOXOs depends on both phosphorylation/14-3-3 binding and intrinsic NES within FOXOs (Brunet et al. 2002). The leucine-rich NES is identifiable by the conserved exportin protein chromosomal region maintenance protein 1 (CRM1), which facilitates nuclear export by interacting with Ran-GTP (Brunet et al. 2002; Van Der Heide et al. 2004).

FOXOs possess substantial disordered domains including the TAD found at the C-terminus of FOXO proteins (Wang et al. 2015). The FOXO TAD is a versatile binding domain that includes two conserved “ΦxxΦΦ” motifs, where “Φ” represents a hydrophobic residue and “x” represents any arbitrary residue (Van Der Heide et al. 2004; Wang et al. 2012b). These regions are crucial for the interaction with coactivators like CBP/p300, effectively increasing the transactivation potential (Nasrin et al. 2000; Wang et al. 2012b). Interestingly, the first “ΦxxΦΦ” motif, also known as the LxxLL motif, is highly conserved among FOXO1, FOXO3, and FOXO4 but not FOXO6 (Zhao et al. 2001) (Fig. 1A).

## Regulation of FOXO activity

FOXO activity is crucial for maintaining cellular homeostasis in response to various stimuli and is primarily regulated by post-translational modifications (PTMs) through several mechanisms (Table 1) (Eijkelenboom and Burgering 2013; Housley et al. 2009; Klotz et al. 2015; Matsuzaki et al. 2003; Yamagata et al. 2008). These PTMs allow for precise control of FOXO activity by affecting their protein localization, stability, DNA binding affinity, or protein interactions (Brown and Webb 2018). Besides PTMs, the regulation of FOXO activity also occurs at the post-transcriptional level, targeting the stability and translation efficiency of FOXO mRNA (Urbanek and Klotz 2017).

**Table 1:** Post-translational modifications of FOXO proteins

**PTM**	**Upstream regulator**	**DAF-16**	**FOXO1**	**FOXO3**	**FOXO4**	**FOXO6**	**Effect**
Phosphorylation	AKT	Ser78, Thr273, Ser345 (Li et al. 2021; Takahashi et al. 2011)	Thr24, Ser256, Ser319 (Guo et al. 1999; Rena et al. 1999, 2001, 2002)	Thr32, Ser253, Ser315 (Becher et al. 2018; Brunet et al. 1999; Kashii et al. 2000)	Thr32, Ser197, Ser262 (Kops et al. 1999; Matsuzaki et al. 2005b; Takaishi et al. 1999)	Thr26, Ser184 (Jacobs et al. 2003; Kim et al. 2011; van der Heide et al. 2005)	Inhibition
Phosphorylation	SGK	Unclear (Chen et al. 2013; Hertweck et al. 2004; Jones et al. 2009)	Thr24, Ser256, Ser319 (Liu et al. 2000)	Thr32, Ser253, Ser315 (Brunet et al. 2001)			Inhibition
Phosphorylation	ERK			Ser294, Ser344, Ser425 (Yang et al. 2008)			Inhibition
Phosphorylation	CK1		Ser322, Ser325 (Rena et al. 2002)				Inhibition
Phosphorylation	CDK2		Ser249 (Huang et al. 2006)				Inhibition
Phosphorylation	DYRK		Ser329 (Woods et al. 2001)				Inhibition
Phosphorylation	IKK			Ser644 (Hu et al. 2004)			Inhibition
Phosphorylation	JNK		Unclear (Weng et al. 2016)		Thr447, Thr451 (De Ruiter et al. 2001; Essers et al. 2004)		Activation
Phosphorylation	p38			Ser7 (Ho et al. 2012)			Activation
Phosphorylation	MST1		Ser212 (Lehtinen et al. 2006)	Ser207 (Yuan et al. 2009)			Activation
Phosphorylation	CDK1		Ser249 (Yuan et al. 2008)				Activation
Phosphorylation	AMPK			Thr179, Ser399, Ser413, Ser439, Ser555, Ser588 (Greer et al. 2007)			Activation
Acetylation	CBP/p300		Lys242, Lys245, Lys262 (Daitoku et al. 2004; Matsuzaki et al. 2005a)		Lys186, Lys189, Lys408 (Fukuoka et al. 2003)		Inhibition
Deacetylation	SIRT1		Unclear (Motta et al. 2004)	Unclear (Brunet et al. 2004; Motta et al. 2004)	Unclear (Motta et al. 2004)		Inhibition
Deacetylation	SIRT1	Unclear (Mouchiroud et al. 2013; Tissenbaum and Guarente 2001)	Lys242, Lys245, Lys262 (Daitoku et al. 2004)	Lys242, Lys245 (Brunet et al. 2004)	Lys186, Lys189, Lys408 (Kobayashi et al. 2005)		Activation
Poly-ubiquitination	SKP2		Ser256 (Huang et al. 2005)				Inhibition
Poly-ubiquitination	CHIP		Ser256 (Li et al. 2009)				Inhibition
Poly-ubiquitination	COP1		Thr32, Ser256, Thr319 (Kato et al. 2008)				Inhibition
Poly-ubiquitination	MDM2			Ser294, Ser344, Ser425 (Yang et al. 2008)			Inhibition
Mono-ubiquitination	MDM2				Unclear (Brenkman et al. 2008)		Activation
Deubiquitination	USP7				Lys199, Lys211 (van der Horst et al. 2006)		Inhibition
Methylation	PRMT1		Arg248, Arg250 (Yamagata et al. 2008)				Activation
Methylation	PRMT6			Arg188, Arg249 (Choi et al. 2019)			Activation
Methylation	SET9			Lys270 (Xie et al. 2012)			Inhibition
O-GlcNAcylation	OGT		Unclear (Housley et al. 2008, 2009; Kuo et al. 2008)				Activation
O-GlcNAcylation	OGT			Ser284 (Shin et al. 2018)			Inhibition

### Phosphorylation

Phosphorylation is the most prominent PTM that regulates FOXO activity. Some protein kinases, including AKT (Brunet et al. 1999; Kops et al. 1999; Matsuzaki et al. 2005b; Rena et al. 1999), SGK (Brunet et al. 2001; Liu et al. 2000), ERK (Yang et al. 2008), CK1 (Rena et al. 2002), CDK2 (Huang et al. 2006), DYRK (Woods et al. 2001), IKK (Hu et al. 2004), can phosphorylate FOXO transcription factors, resulting in the creation of a docking site for 14-3-3 proteins, which translocate FOXO proteins into the cytoplasm and impede their reentry into the nucleus, thereby inhibiting FOXO transcriptional activity. In most cases, the phosphorylation modification of FOXO drives its localization to the cytoplasm, but a few phosphorylated forms promote FOXO entry into the nucleus. Under oxidative stress, intracellular reactive oxygen species (ROS) trigger FOXO phosphorylation and their translocation from the cytoplasm to the nucleus via activation of c-Jun amino-terminal kinase (JNK) (Essers et al. 2004; Weng et al. 2016). This process induces stress defense genes and extends the lifespan of *Drosophila* (Wang et al. 2005). p38-mediated phosphorylation of FOXO3 at Ser7 promotes its nuclear relocalization in response to doxorubicin (Ho et al. 2012). MST1 (Lehtinen et al. 2006; Yuan et al. 2009) and CDK1 (Yuan et al. 2008) phosphorylate FOXOs at specific serine sites, disrupting their interaction with 14-3-3 proteins and leading to nuclear translocation, followed by the induction of cell death in neurons. However, AMP-activated protein kinase (AMPK)-mediated phosphorylation does not affect the subcellular localization of FOXO3 but rather activates the expression of target genes by promoting the interaction of FOXO3 with other cofactors (Greer et al. 2007). In line with this, pharmacological or genetic activation of AMPK activates FOXO3 and its downstream pro-apoptotic target gene PUMA, which in turn suppresses the metastatic progression of pancreatic ductal adenocarcinoma (Nagarajan et al. 2017).

FOXO proteins, known as tumor suppressors, are frequently deregulated in human cancer, primarily through AKT-mediated phosphorylation (Dansen and Burgering 2008; Hennessy et al. 2005). High levels of AKT-phosphorylated FOXO proteins are associated with reduced overall survival in various cancers (Hornsveld et al. 2018; Zhang et al. 2009). Inactivation of FOXO1 removes its ability to inhibit RUNX2, favoring prostate cancer progression (Zhang et al. 2011a). AKT-mediated FOXO4 phosphorylation downregulates ANXA8 expression, leading to the epithelial-to-mesenchymal transition process and tumor metastasis in cholangiocarcinoma (Lee et al. 2009). FOXO proteins can also be inactivated by IKK-mediated phosphorylation in breast cancer (Hu et al. 2004) and leukemia (Chapuis et al. 2010), independently of AKT. ERK phosphorylates FOXO3 at Ser294, Ser344, and Ser425, leading to FOXO3 degradation via an MDM2-mediated ubiquitin-proteasome pathway, promoting cell proliferation and tumorigenesis (Yang et al. 2008). Accordingly, pharmacological activation of FOXO3 has been shown to restore normal physiological conditions and reprogram ovarian and breast cancer cells into non-cancerous cells (Hu et al. 2014). In breast and ovarian cancer, active FOXO proteins are associated with a good prognosis, while their inhibition is linked to poor survival (Fei et al. 2009; Habashy et al. 2011; Jiang et al. 2013).

### Acetylation

Acetylation serves as an additional regulatory mechanism to fine-tune FOXO activity. Acetylation of FOXOs is predominantly regulated by histone acetyltransferases (HATs) such as CBP/p300, while its deacetylation is mediated by histone deacetylases (HDACs) including the sirtuins family members (Dansen et al. 2009; Motta et al. 2004; van der Heide and Smidt 2005). Acetylation sites, such as Lys242, Lys245, and Lys262 in FOXO1, are typically located in the Forkhead DBD of FOXO proteins. Acetylation at these sites of FOXOs has been reported to attenuate their DNA binding affinity and transcriptional activity (Daitoku et al. 2004; Matsuzaki et al. 2005a). However, it is inconclusive whether acetylation modifications ultimately lead to an increase or decrease in FOXO activity (Kobayashi et al. 2005; van der Heide and Smidt 2005). For example, the NAD-dependent deacetylase SIR2 modulates the longevity of *C. elegans* through activation of FOXO signaling (Mouchiroud et al. 2013; Tissenbaum and Guarente 2001), whereas its mammalian ortholog SIRT1 deacetylates and represses the activity of FOXO3 and other Forkhead factors in mammals (Motta et al. 2004). Additionally, acetylation has been shown to enhance FOXO target genes that induce apoptosis, while attenuating the expression of target genes associated with cell-cycle arrest and anti-oxidative stress (Brunet et al. 2004). These findings raise a possibility that acetylation may regulate FOXO activity in a target-specific manner (Calissi et al. 2021; van der Heide and Smidt 2005).

Acetylation of FOXO proteins has been shown to enhance their tumor-suppressive function. For instance, acetylated FOXO1 binds ATG7, an E1-like protein in the cytosol, leading to autophagic cell death in human colon cancer tissue (Zhao et al. 2010). Additionally, CBP/p300-mediated acetylation of FOXO1 induces apoptosis and inhibits pancreatic tumor growth (Pramanik et al. 2014). Conversely, deacetylation of FOXOs by SIRTs or HDACs has been linked to cancer progression and metastasis. SIRTs repress FOXO acetylation to promote cancer survival and metastasis by neutralizing oxidative stress (Kenny et al. 2017; Papa and Germain 2014). Four and a half LIM2 (FHL2) suppresses FOXO1 activity by SIRT1-mediated deacetylation, enhancing prostatic tumorigenesis (Yang et al. 2005). HDAC3 facilitates FOXO3 deacetylation and breast cancer metastasis (Zhang et al. 2017a). Furthermore, deacetylation of FOXOs is associated with resistance to chemotherapeutic drugs. For example, SIRT6 promotes resistance to paclitaxel and epirubicin in breast cancer by modulating FOXO acetylation and expression (Khongkow et al. 2013). Cisplatin-resistant cells possess a reduced amount of acetylated FOXO3 compared with their parental cells (Shiota et al. 2010). In agreement with this, treatment with SIRT inhibitors (Kojima et al. 2008) or siRNA (Liang et al. 2008) sensitizes cisplatin-resistant cells to cisplatin, indicating that acetylation of FOXOs could be a critical target for intervention in cancer progression or therapeutic resistance.

### Ubiquitination

Ubiquitination is a dynamic process that is tightly regulated by the enzymatic activities of E1 ubiquitin-activating enzymes, E2 ubiquitin-conjugating enzymes, and E3 ligases (Komander 2009). FOXOs are substrates for ubiquitination mediated by certain ubiquitin E3 ligases, including S-phase kinase-associated protein 2 (SKP2) (Huang et al. 2005), Hsc70-interacting protein (CHIP) (Li et al. 2009), and constitutive photomorphogenic 1 (COP1) (Kato et al. 2008). These E3 ligases recognize and promote ubiquitin-dependent degradation of FOXOs, thereby repressing FOXO functions. Notably, AKT-dependent phosphorylation of FOXO1 is necessary for its interaction with the E3 ligases, for example, SKP2 and CHIP recognize FOXO1 phosphorylated at Ser256 (Huang et al. 2005; Li et al. 2009), while COP1 binds FOXO1 phosphorylated at multiple sites by AKT (Kato et al. 2008). Similarly, phosphorylation of FOXO3 mediated by ERK recruits another E3 ubiquitin ligase, MDM2, and leads to subsequent FOXO3 poly-ubiquitination and proteasome-mediated degradation (Yang et al. 2008). Poly-ubiquitination typically leads to protein degradation via the proteasome, while monoubiquitination serves non-degradative functions such as protein-protein interaction and protein trafficking (Magits and Sablina 2022; Sigismund et al. 2004). For example, MDM2 can mediate the mono-ubiquitination of FOXO4, which in turn facilitates the activation of FOXO4 in response to oxidative stress (Brenkman et al. 2008). On the other hand, the deubiquitinating enzyme ubiquitin-specific protease (USP7) deubiquitinates FOXO4, thereby inhibiting FOXO activity through nuclear exclusion (van der Horst et al. 2006). It should be noted that different PTMs may compete at the same site, such as acetylation and ubiquitination modifications both on the ɛ-amino group of lysine residues, while SIRT1-mediated FOXO3 deacetylation drives the opening of lysine residues (Lys242, Lys259, Lys290, and Lys569), which facilitates FOXO3 polyubiquitination and subsequent degradation (Wang et al. 2012a).

### Methylation

Protein methylation modifies nitrogen atoms in amino acids such as Lys, Arg, His, Ala, and Asp, with particular emphasis on Lys and Arg residues (Lee et al. 2005). Protein arginine methyltransferase PRMT1 has been found to methylate FOXO1 at Arg248 and Arg250, effectively blocking AKT-mediated phosphorylation of Ser253 and nuclear exclusion of FOXO1 (Yamagata et al. 2008). Accumulation of β-amyloid triggers neurodegeneration and boosts PRMT1-mediated arginine methylation of FOXO3, resulting in increased nuclear accumulation of FOXO3 (Sanphui and Biswas 2013; Selkoe 2001). Nuclear FOXO3 directly binds to the promoter of BIM, a pro-apoptotic member of the BCL-2 family, increasing its expression and ultimately leading to neuronal apoptosis in Alzheimer’s disease (Sanphui and Biswas 2013). Intriguingly, arginine methylation of FOXOs by PRMT1 has also been observed in nematodes and insects, acting as a fascinating ‘anti-aging’ modification by impeding AKT-mediated phosphorylation of FOXO, ultimately leading to lifespan extension (Takahashi et al. 2011; Zhang et al. 2017b). PRMT6 methylates and activates FOXO3 at Arg188 and Arg249, enhancing protein degradation and autophagic pathways in skeletal muscles, with potential implications for preventing and intervening in muscle atrophy (Choi et al. 2019). These findings suggest arginine methylation may serve as a positive regulator of FOXO activity. Nevertheless, lysine methylation of FOXO3 at Lys270 by SET9 methyltransferase reduces its transcriptional activity, inhibiting FOXO3-mediated BIM expression and oxidative stress-induced neuronal cell death (Xie et al. 2012). Interestingly, SET9-mediated FOXO3 methylation at Lys271 does not affect its subcellular localization but decreases FOXO3 protein stability (Calnan et al. 2012).

### O-GlcNAcylation

O-GlcNAcylation is a reversible PTM that involves adding or removing a sugar called N-acetylglucosamine (GlcNAc) to or from serine or threonine residues, which is controlled by the dynamic interplay between O-GlcNAc transferase (OGT) and O-GlcNAcase (OGA) (Hart et al. 2007; Sheikh et al. 2021). In *C. elegans*, *ogt-1* mutant animals have a shorter lifespan, while *oga-1* mutation extends their lifespan (Love et al. 2010; Rahman et al. 2010). However, it remains unclear whether DAF-16/FOXO is directly O-GlcNAcylated in worms. In *Drosophila*, O-GlcNAc modification of FOXO influences the autophagy pathway and regulates growth and longevity (Akan et al. 2021; Park et al. 2015). In mammals, O-GlcNAcylation of FOXO1 enhances its transcriptional activity (Kuo et al. 2008), serving as a glucose sensor in the liver to mediate FOXO1-dependent transcription of gluconeogenesis and stress response genes (Housley et al. 2008). Further investigation reveals the mechanism by which the coactivator PGC-1α interacts with OGT to target FOXO1, resulting in enhanced O-GlcNAcylation and increased transcriptional activity (Housley et al. 2009). Another study has indicated that O-GlcNAcylation of FOXO4 results in enhanced transcriptional activity and provides cell survival signaling in response to acute oxidative stress (Ho et al. 2010). In the context of tumorigenesis, O-GlcNAcylation at FOXO3 Ser284 abrogates its tumor suppressor activity by activating the MDM2-p53-p21 signaling, leading to accelerated pancreatic cancer cell growth (Shin et al. 2018).

### Post-transcriptional modifications of FOXO proteins

MicroRNAs (miRNAs) are endogenous small noncoding RNAs that fine-tune gene expression by promoting transcriptional degradation or repressing translation, mostly via the 3′-untranslated region (UTR) of target mRNA (Bartel 2009). Emerging evidence has begun to elucidate the interaction between miRNAs and FOXOs. miR-71 promotes longevity in *C. elegans* by facilitating DAF-16/FOXO activity in the intestine (Boulias and Horvitz 2012). miR-34, miR-35, and let-7 family members confer resilience to environmental stresses and toxicity in *C. elegans* by modulating DAF-16/FOXO levels (Isik et al. 2016; Li et al. 2020; Wang et al. 2022). Several miRNAs have been discovered to act as regulators of FOXO expression in various cancer types (Duwe et al. 2023; Liu et al. 2018; Urbanek and Klotz 2017). For instance, miR-27a promotes cell proliferation and epithelial-mesenchymal transition by downregulating FOXO1 in obesity-associated liver cancer and ovarian cancer (Sun et al. 2015; Zhang et al. 2019). miR-629 boosts proliferation and invasion in pancreatic cancer by targeting FOXO3 (Yan et al. 2017). Additionally, oncogenic miR-664 suppresses FOXO4 expression in osteosarcoma cells, resulting in increased cancer cell proliferation (Chen et al. 2015).

In the context of stem cell behaviors, miR-182 serves as a FOXO1 inhibitor to antagonize proliferation and differentiation of MSCs and osteoblasts, with a subsequent negative effect on osteogenesis (Kim et al. 2012). Estrogen deficiency reduces FOXO1 activity via miR-705 post-transcriptional regulation, causing oxidative damage in bone marrow-derived mesenchymal stem cells (BMSCs) and impaired osteogenic differentiation (Liao et al. 2016). Sun et al. recently found a potential therapy for cartilage repair and osteoarthritis (OA) using extracellular vesicles (EVs) from TGFβ3-preconditioned BMSCs. miR-455 enriched in these EVs promotes OA alleviation and cartilage regeneration by stimulating the SOX11-FOXO signaling (Sun et al. 2022). Inhibition of miR-195 in skeletal muscle-derived stem/progenitor cells (SkMDS/PCs) increases FOXO3 expression, supporting SkMDS/PCs maintenance through antioxidant gene activation (Gopinath et al. 2014; Nowaczyk et al. 2022). A similar miRNA-FOXO correlation is also reported in goat muscle cells (Xu et al. 2021).

RNA modification regulates the mRNA fate of FOXOs, particularly through N6-methyladenine (m6A) methylation, the most prevalent type of RNA methylation in eukaryotic mRNAs (Boo and Kim 2020; Meyer and Jaffrey 2014). In ovarian granulosa cells, m6A methylation affects FOXO signaling, with hyper m6A methylation downregulating FOXO6 mRNA in aged cells, potentially contributing to ovarian aging (Liu et al. 2022). RNA-binding proteins like the Hu antigen R (HuR) and Quaking (QKI) also influence the post-transcriptional regulation of FOXO expression (Guo et al. 2014; Li et al. 2013; Yu et al. 2014). HuR interacts with FOXO1 mRNA and stabilizes its expression, thereby augmenting 5-fluorouracil (5-FU)-induced apoptosis in breast cancer cells (Li et al. 2013). Conversely, QKI destabilizes FOXO1 mRNA and contributes to the oncogenesis and progression of breast carcinoma (Yu et al. 2014). These findings suggest that modulating FOXO expression at the post-transcriptional level could offer a promising strategy for cancer therapy.

## Role of FOXOs in regulating stem cell fate decision

The decision of stem cells to maintain pluripotency or differentiate is a finely regulated process influenced by various factors. Both self-renewal and quiescence maintenance are essential mechanisms for retaining pluripotency in stem cells. Self-renewal involves creating identical copies of stem cells via cell division, while quiescence maintenance regulates cell cycle progression to protect pluripotency. The exact mechanisms that regulate pluripotency versus differentiation can vary depending on the specific type of stem cell and environmental factors, and continue to be an area of active research in stem cell biology. In the following sections, we discuss in detail the roles of FOXOs in stem cell self-renewal, quiescence maintenance, and differentiation.

### Self-renewal of pluripotent stem cells

Self-renewal plays a vital role in maintaining the pluripotency of stem cells. It enables stem cells to divide and generate more identical stem cells, allowing for a continuous supply of undifferentiated cells. By self-renewing, stem cells can maintain their ability to differentiate into various cell types while simultaneously replenishing the stem cell population. This self-renewal process helps sustain the pool of pluripotent stem cells and ensures their ongoing potential for embryonic development and tissue regeneration. FOXO transcription factors act as critical regulators of stem cell self-renewal and pluripotency in various species.

*Hydra vulgaris*, a freshwater radial-symmetric polyp of the phylum Cnidaria, exhibits biological immortality due to the infinite self-renewal capacity of stem cells, including interstitial stem cells (i-cells) and ectodermal/endodermal epithelial stem cells (Bosch 2009). The i-cells exhibit multipotent properties whereas the epithelial cell lineages represent unipotent stem cells, enabling them to undergo differentiation into multiple distinct cell types including nematocyte, nerve cell, gland cell, and germ line (Hemmrich et al. 2012). *Hydra* has a single FOXO gene that was previously believed to contribute to stress resistance (Bridge et al. 2010). Further studies demonstrate that *Hydra* FOXO is expressed in all three stem cell lineages and contributes to their continuous self-renewal (Boehm et al. 2012; Hemmrich et al. 2012). Overexpression of FOXO in i-cells leads to an increase of proliferation in both stem cell and progenitor cells, furthermore, it imparts stemness to terminally differentiated cells. Conversely, silencing of FOXO represses stem cell gene networks and expedites terminal differentiation in the epithelial stem cells (Boehm et al. 2012). Like other bilaterian animals, *Hydra* FOXO function is negatively regulated by the PI3K/AKT signaling; however, its activity in interstitial lineage cells appears unaffected under dietary restriction, probably due to the absence of insulin/IGF-1 receptors and a corresponding response to nutrient conditions in the interstitial cells (Bridge et al. 2010).

*Hofstenia miamia*, commonly known as the three-banded panther worm, belongs to a deep-diverging bilaterian Xenacoelomorpha lineage. The acoel worm has gained attention for its ability to regenerate tissue via a population of adult pluripotent stem cells, called neoblasts (Gehrke et al. 2019; Srivastava et al. 2014). To investigate the embryonic origins of these adult stem cells, Kimura et al. systematically performed photo-conversion on each cell of the early embryo, creating a comprehensive fate map at the eight-cell stage. The authors identified a specific pair of cells at the sixteen-cell stage that give rise to neoblast-like cells, which contribute to tissue regeneration and homeostatic turnover. Through further analysis using single-cell transcriptome profiling, a specific set of genes, including *foxO* and *tbx*, emerge as potentially significant regulators of neoblast formation during embryonic development. Notably, *foxO* RNAi animals exhibit diminished expression of *piwi-1*, a master factor that is required for neoblast self-renewal (Kimura et al. 2022). These discoveries emphasize the essential role of FOXOs in the process of stem cell self-renewal.

In addition to invertebrates, FOXO family members are important regulators of ESC pluripotency in mammals. The pluripotency of ESCs is maintained by the interplay of specific transcription factors, such as OCT4, SOX2, and NANOG, creating a self-regulatory feedback loop that activates genes encoding essential pluripotency factors, while simultaneously repressing genes associated with differentiation (Boyer et al. 2005). In human ESCs (hESCs), FOXO1 promotes pluripotency by directly binding and activating OCT4 and SOX2 genes, while the absence of FOXO1 leads to the spontaneous differentiation of hESCs, even when maintained under pluripotent self-renewal conditions (Zhang et al. 2011b). A similar role of FOXO1 in maintaining pluripotency is also found in mouse ESCs (Zhang et al. 2011b). To maintain intracellular balance in ESCs, damaged organelles and toxic proteins must be promptly cleared, while the total biomass associated with their rapid proliferation rate needs to be synthesized. This delicate task is achieved through the coordinated efforts of autophagy and the proteasome system, important components of the cellular quality control machinery (Buckley et al. 2012; Liu et al. 2016). Notably, FOXO1 directly regulates core autophagy genes, ensuring high autophagic flux for ESC pluripotency (Liu et al. 2017). Similarly, FOXO4 is responsible for enhancing proteasome assembly and activity, which are fundamental aspects of ESC identity and pluripotency maintenance (Vilchez et al. 2012). This function of FOXO1 in maintaining self-renewal and pluripotency appears to be conserved across various species, from invertebrates to mammals.

### Quiescence maintenance of adult stem cells

Adult stem cells (ASCs) serve as a cellular reservoir responsible for tissue homeostasis and regeneration after injuries (de Morree and Rando 2023). ASCs in tissues with high cell turnover, such as the gut epithelium and blood, continuously proliferate and replenish the lost cells, ensuring tissue renewal and maintenance (Barker et al. 2010). Alternatively, long-lived ASCs can enter a quiescent state to preserve potency and protect against premature depletion or adverse conditions. Quiescent ASCs, characterized by reversible mitotic arrest and reduced metabolic activity, are well established in certain tissues, including skeletal muscle, brain, and bone marrow (de Morree and Rando 2023; Li and Bhatia 2011). The quiescent state plays a vital role in the long-term maintenance of the ASC pool and should be properly regulated. FOXOs have been demonstrated to play a crucial role in maintaining the proper functioning and regenerative capacity of ASCs across diverse tissue types (Gopinath et al. 2014; Ro et al. 2013; Tothova and Gilliland 2007).

Myogenic stem cells, also known as satellite cells (SCs), are present in skeletal muscles in a quiescent state, which is essential for maintaining the SC pool and preserving their proliferative potential. When muscle fibers are injured, SCs activate and undergo self-renewal to provide a cell source for muscle regeneration (Charge and Rudnicki 2004; Yin et al. 2013). In skeletal muscles, FOXO3 is highly expressed and active in quiescent SCs rather than in their activated counterparts (Gopinath et al. 2014). Depletion of FOXO3 hinders activated SCs from re-entry into the quiescent state, resulting in exhaustion of the SC pool and impaired muscle regeneration (Gopinath et al. 2014). FOXO3 preserves SC quiescence by modulating Notch signaling, which has been associated with the promotion or maintenance of SC quiescence (Bjornson et al. 2012; Mourikis et al. 2012; Wen et al. 2012). Juvenile mice lacking FOXOs in their SCs fail to enter quiescence and have high expression of myogenic-differentiation-related genes such as *Myogenin*, along with downregulation of stemness- and quiescence-related genes such as *Notch3*. This failure endures throughout the lifespan and is further exacerbated in advanced age (Garcia-Prat et al. 2020). Aging leads to a progressive loss of SC quiescence due to intrinsic and niche-related alterations (Chakkalakal et al. 2012; Sousa-Victor et al. 2014). In geriatric SCs, niche-derived IGF1-dependent AKT signaling is more active than in young cells, leading to a decline in the quiescent state by inhibiting FOXO signals. This contributes to muscle regenerative failure in geriatric mice (Garcia-Prat et al. 2020). Therefore, strategies aimed at counteracting AKT and boosting FOXO activity offer a potential therapeutic approach to restore SC quiescence and improve the regenerative capacity of skeletal muscle.

Quiescent neural stem cells (NSCs) are generated early at embryonic stages and reside in specific regions of the adult brain, such as the hippocampus and the subventricular zone (Fuentealba et al. 2012; Furutachi et al. 2015). When the nervous system is damaged, these quiescent NSCs can be activated to replace damaged or lost neurons and glial cells. The quiescent state safeguards NSC homeostasis by preserving its stemness and preventing depletion (Llorens-Bobadilla et al. 2015; Otsuki and Brand 2020). FOXOs play a critical role in enforcing NSC quiescence and self-renewal control. The absence of FOXO1, FOXO3, and FOXO4 in mice initially leads to increased brain size and transient hyper-proliferation of NSCs, but ultimately depletes the self-renewing NSCs in older adult mice (Paik et al. 2009). Knockout of FOXO3 alone, either in the whole animal or specifically in the brain, is sufficient to reduce the multipotency and self-renewal capacity of adult NSCs (Renault et al. 2009). Microarray-based transcriptional profiling reveals that FOXO3 regulates the NSC pool by activating genes involved in quiescence, such as *p27*^*KIP1*^ and *Cyclin G2* (Renault et al. 2009). FOXO3 also shares common binding sites with the proneuronal bHLH transcription factor ASCL1 in neural progenitor cells, helping to preserve the NSC pool by restraining ASCL1-dependent neurogenesis (Webb et al. 2013). Excessive ROS causes stem cell decline and drives them out of quiescence, while FOXO3 helps counteract oxidative stress and preserve the long-term proliferative potential of NSCs (Ludikhuize and Rodriguez Colman 2021; Rossi et al. 2008; Yeo et al. 2013). Additionally, FOXOs preserve the NSC pool by activating the autophagy network (Audesse et al. 2019) and coordinating metabolic programs (Yeo et al. 2013). Brain aging is linked to cognitive impairment and increased risk of neurodegeneration (Nicaise et al. 2020). NSC depletion due to a high-fat diet may contribute to cognitive decline in age-related brain diseases by inhibiting FOXO activity (Kuhn et al. 1996; Renault et al. 2009). The stimulation of aged NSCs with young NSC-derived exosomes rescues FOXO activation and reinstates the equilibrium between proliferating and senescent NSCs in the hippocampus, thereby counteracting high-fat diet-dependent impairment of adult hippocampal neurogenesis in mice (Natale et al. 2022). These findings underscore the therapeutic potential of extracellular vesicles in preventing both physiological and pathological cognitive decline.

Hematopoietic stem cells (HSCs), a type of multipotent stem cell found in the bone marrow and umbilical cord blood, are responsible for the replenishment and renewal of the blood and immune system (Orkin and Zon 2008). The majority of HSCs are maintained in an undifferentiated quiescent state within a bone marrow niche, and interruption of this dormant state disturbs HSC function (Arai et al. 2004; Rossi et al. 2012). FOXOs have been shown to have a significant impact on HSC maintenance (Miyamoto et al. 2007, 2008; Tothova et al. 2007; Yalcin et al. 2008). HSCs derived from FOXO1/3/4-deficient mice have an aberrant increase in cell cycling, apoptosis, and ROS levels, resulting in impaired long-term repopulating activity in vivo (Tothova et al. 2007). Notably, ablation of FOXO3 alone is adequate to drive HSCs to enter the cell cycle, resulting in HSC depletion and less resistance to myelotoxic drugs (Miyamoto et al. 2007). Likewise, Yalcin et al. hold the view that FOXO3 is the principal active FOXO in HSCs and regulates oxidative stress by modulating the expression of ATM (Yalcin et al. 2008). Loss of FOXO3 leads to ROS accumulation, thereby activating the p53/p21 pathway and causing exit from quiescence and G2/M arrest (Yalcin et al. 2008). FOXO3 retains a pro-autophagy gene program in aging HSCs, crucial for their survival in response to metabolic stress (Warr et al. 2013). As HSCs age, their function decreases (Rossi et al. 2005; Sudo et al. 2000). The microRNA-212/132 cluster, abundant in HSCs, is upregulated during aging. HSCs lacking miR-132 and miR-212 exhibit elevated FOXO3 expression and enhanced quiescence, which contributes to improved engraftment potential, reduced apoptosis, and enhanced resistance to inflammatory stress (Mehta et al. 2015). This suggests the potential use of miRNA antagonists to enhance functions in aged HSCs.

**Fig. 2:**
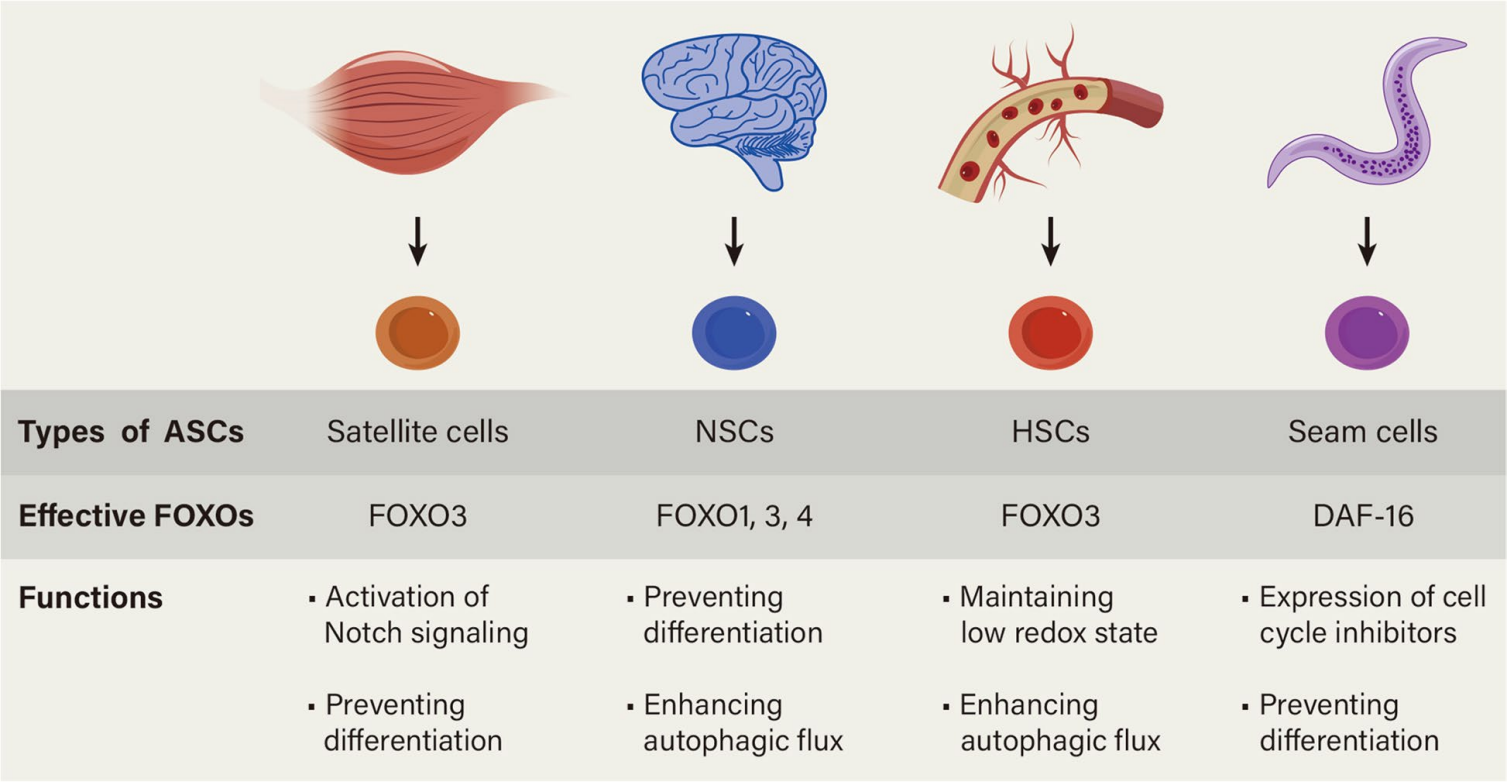
The role of FOXOs in maintaining quiescence in adult stem cells. The quiescent state is crucial for the long-term maintenance of the adult stem cell pool, and DAF-16/FOXO is essential for preserving stem cell quiescence, a trait that seems to be conserved across species

The above findings indicate that FOXOs, particularly FOXO3, play a crucial role in maintaining the quiescence of ASCs, which is reminiscent of the role of DAF-16 in *C. elegans* during the stress-resistant stage known as dauer. Though *C. elegans* has a short lifespan of 2–3 weeks, it exhibits life history plasticity. Under favorable conditions, the animals transition rapidly through four continuous larval stages to adulthood. However, in adverse conditions like food scarcity and population congestion, they enter a quiescent and stress-resistant stage called dauer (Fielenbach and Antebi 2008). Interestingly, the longevity gene DAF-16, the sole *C. elegans* FOXO ortholog, primarily functions in dauer life history (Libina et al. 2003; Lin et al. 1997; Ogg et al. 1997). During dauer, DAF-16/FOXO mediates quiescent cell fate through activation of the cyclin-dependent kinase inhibitor CKI-1 as well as stress resistance pathways (Baugh and Sternberg 2006; Isik et al. 2016; Lamitina and Strange 2005). It’s important to note that stem cells in dauer larvae must maintain multipotency for an extended period to resume development quickly once favorable conditions ensue (Karp and Greenwald 2013; Liu and Ambros 1991). Karp et al. found that DAF-16/FOXO maintains or restores multipotency in vulval precursor cells (VPCs) of dauer larvae by inhibiting EGFR and Lin12-Notch signaling pathways (Karp and Greenwald 2013). DAF-16/FOXO also promotes multipotency in epidermal stem cells (seam cells) during dauer by upregulating *lin-41*, a heterochronic gene that prevents premature differentiation (Wirick et al. 2021). Additionally, DAF-16/FOXO regulates the germline stem cell pool in a cell-nonautonomous manner (Qi et al. 2012; Qin and Hubbard 2015). Thus, DAF-16/FOXO is crucial for maintaining stem cell quiescence, which appears to remain conserved in mammals as mentioned above (Fig. 2).

### Context-dependent regulation of stem cell differentiation

FOXO transcription factors play a significant role in stem cell differentiation due to their ability to modulate gene expression and cellular processes involved in this process. Understanding the precise mechanisms by which FOXO factors impact stem cell differentiation holds promise for advancing regenerative medicine and therapeutic applications. However, the effect of FOXOs on differentiation is complex and context-dependent, with the potential for both promotion and inhibition depending on specific transcriptional targets.

#### Neurogenesis

Neurogenesis, the generation of new neurons from ESCs or NSCs, plays a crucial role in nervous system development, cognitive functions, and neural repair (Li and Guo 2021; Obernier and Alvarez-Buylla 2019). Studies have focused on understanding the roles of different FOXO members in neurogenesis.

FOXO1 is expressed in NSCs in the neurogenic subventricular zone, with its expression decreasing during early neurogenesis (Kim et al. 2015). When activated, FOXO1 inhibits NSC differentiation into neurons, while FOXO1 deficiency enhances neuronal differentiation, indicating its inhibitory role in neurogenesis. FOXO1 interacts with the CSL transcription factor to activate the Notch target gene HES1, which is crucial for NSC maintenance and limiting the spread of differentiation (Hitoshi et al. 2002; Ishibashi et al. 1994; Kageyama and Ohtsuka 1999). Similarly, FOXO3 helps preserve the NSC pool by restraining proneuronal bHLH transcription factor ASCL1-dependent neurogenesis (Webb et al. 2013), with FOXO3 and ASCL1 being notably concentrated at the enhancers of genes involved in neurogenic pathways, including DLL1 and HES6, which are critical for ASCL1-dependent neurogenesis (Artavanis-Tsakonas et al. 1999; Castro et al. 2006). This aligns with the observation that PTEN loss or AKT activation in NSCs, which leads to FOXO inhibition, promotes a significant increase in neurogenesis (Gregorian et al. 2009).

On the other hand, FOXO4 has been implicated in the neural differentiation of ESCs. Loss of FOXO4 reduces neural lineage differentiation potential and promotes trophoblast or keratinocyte differentiation instead (Vilchez et al. 2013). This may be attributed to decreased expression of PAX6, an important ectodermal transcription factor regulating neuronal gene activation and repression of mesodermal/endodermal genes (Thakurela et al. 2016). Considering the vital roles of FOXO1 and FOXO3 in regulating NSC maintenance, it is speculated that FOXO4 may be required during embryonic stages of neural development, while FOXO1 and FOXO3 are necessary for NSC maintenance and regeneration in adulthood. These findings highlight the distinct roles played by different FOXO family members in neurogenesis. Further research in this area can provide valuable insights into the underlying mechanisms and potential therapeutic interventions for neural regeneration and repair.

#### Myogenesis

Muscle development involves several steps, starting from the formation of myogenic precursors to the differentiation of myoblasts (McKinsey et al. 2001). Insulin-like growth factors (IGFs) promote myoblast differentiation through the PI3K/AKT signaling pathway (Coolican et al. 1997; Jiang et al. 1999; Tureckova et al. 2001). FOXO1, a key target of AKT, plays a significant role in mediating myoblast differentiation in response to IGF signaling (Hribal et al. 2003). Constitutively active mutant FOXO1 inhibits myoblast differentiation, while dominant-negative mutant FOXO1 partially rescues impaired differentiation induced by a PI3K inhibitor (Hribal et al. 2003; Wu et al. 2008). FOXO1 also interacts with and activates Notch signaling, inhibiting MyoD-dependent myoblast differentiation and fiber-type specification (Kitamura et al. 2007; Kuroda et al. 1999).

In contrast, FOXO3, alongside PAX3/7, activates MyoD transcription, promoting the differentiation of satellite cells (Hu et al. 2008). This cooperative activation allows for precise regulation of MyoD expression and myogenic potential, complementing the inhibitory effects of FOXO1. Additionally, FOXO4 inhibits smooth muscle cell differentiation by interacting with and inhibiting myocardin, a transcriptional coactivator of smooth muscle genes (Liu et al. 2005). Overall, different FOXO members have distinct roles in myogenesis, contributing to the complexity of muscle development processes.

#### Osteogenic differentiation

Osteogenic differentiation, the process of transforming mesenchymal stem cells (MSCs) into osteoblasts, is carefully regulated by several factors including runt‐related transcription factor 2 (RUNX2), β‐catenin, alkaline phosphatase (ALP), activating transcription factor 4 (ATF4), and osteocalcin (OCN) (Chen et al. 2019). Initially, RUNX2 triggers the expression of important bone matrix protein genes in early progenitors, determining the differentiation of MSCs into the osteoblastic lineage (Komori 2010). Studies suggest that FOXOs act as upstream regulators of RUNX2 during osteogenic differentiation. In FOXO1/3/4-deficient mice, MSCs show reduced RUNX2 expression and impaired osteogenic differentiation potential (Ambrogini et al. 2010). Knocking down FOXO1 or FOXO3 in early progenitors leads to decreased RUNX2 upregulation, while overexpression of these factors results in elevated RUNX2 expression (Siqueira et al. 2011; Teixeira et al. 2010). Additionally, FOXO1 directly interacts with the promoter of RUNX2 and regulates its expression (Siqueira et al. 2011; Teixeira et al. 2010). Together, FOXO1/3 may play a role in initiating the differentiation of MSCs into early progenitors by upregulating RUNX2 expression.

β‐catenin plays a crucial role in the commitment of early progenitors to osteoblast precursors through Wnt/TCF signal transduction (Glass et al. 2005; Hu et al. 2005; Rodda and McMahon 2006). However, FOXO activation attenuates Wnt signaling by competing with TCF for β‐catenin, thereby inhibiting osteoblastic differentiation of uncommitted progenitors (Almeida et al. 2007). Conversely, mice lacking FOXO1, 3, and 4 exhibit an increased number of committed osteoblast precursors by unleashing β‐catenin/TCF activation (Iyer et al. 2013). Therefore, FOXOs may hinder osteogenesis by redirecting β‐catenin from TCF to FOXO-mediated transcription at the early stage of osteogenic lineage commitment.

The maturation of committed osteoblast precursors is characterized by the expression and activity of ALP, ATF4, and OCN (Neve et al. 2011). FOXO1 and FOXO3 act as upstream regulators of ALP, promoting mineralization by supplying inorganic phosphate through pyrophosphate hydrolysis (Ambrogini et al. 2010; Siqueira et al. 2011; Teixeira et al. 2010). FOXO1 also enhances mineralization by interacting with ATF4 to boost protein synthesis and oxidative stress resistance (Rached et al. 2010b). OCN helps balance the process of osteogenesis by promoting bone formation and preventing excessive mineralization (Ducy et al. 1996; Komori 2020). FOXO1 negatively regulates OCN availability through a two-step process, suppressing OCN expression by binding to its promoter and inhibiting OCN bioactivity by promoting γ-carboxylation (Rached et al. 2010a; Yang et al. 2011).

In summary, FOXOs facilitate osteogenesis in early progenitors and mature osteoblasts while impeding it in committed osteoblast precursors. Their stage-specific functions involve interactions with different factors, such as RUNX2 in early progenitors, β‐catenin in osteoblast precursors, and ALP/ATF4/OCN in mature osteoblasts, throughout the process of osteogenic differentiation.

#### Chondrogenic differentiation

MSCs can also differentiate into chondrocytes when exposed to specific growth factors and signaling molecules, with TGF-β being the most prominent one (Oka et al. 2007). This exposure leads to changes in MSC shape and activation of genes associated with chondrogenic transcription factors, such as SOX9, as well as cartilage extracellular matrix components like type II collagen (COL2) and aggrecan (ACAN) (Bell et al. 1997; Ikegami et al. 2011; Sekiya et al. 2000).

Recent studies have demonstrated that FOXO1 expression and activity increase during TGF-β-induced chondrogenic differentiation (Kurakazu et al. 2019). FOXO1, in turn, promotes the expression of COL2 and ACAN and induces cell-cycle arrest in the G0/G1 phase via p21, a cyclin-dependent kinase inhibitor involved in chondrogenic differentiation (Negishi et al. 2001). FOXO3 cooperates with RUNX1 to promote both early and terminal stages of chondrogenesis, leading to the upregulation of specific genes, including SOX9 and COLX, which are targeted by both FOXO3 and RUNX1 (Yuan et al. 2022). The use of a specific inhibitor of FOXO1 and FOXO3, called AS1842856, completely inhibits chondrogenic differentiation (Sharieh et al. 2020), highlighting the significance of FOXO1 and FOXO3 in regulating the chondrogenic differentiation process in MSCs. Overall, these findings shed light on the complex regulatory mechanisms underlying chondrogenic differentiation and provide potential targets for therapeutic interventions.

#### Adipogenesis

Adipogenesis is a complex process that occurs in two stages: commitment of MSCs to a preadipocyte fate, followed by terminal differentiation into mature adipocytes (Cristancho and Lazar 2011). The involvement of FOXO1 in adipogenesis has been a topic of debate in previous studies. Munekata et al. conducted experiments using FOXO1-siRNA in mouse 3T3-L1 preadipocytes and observed that silencing FOXO1 hindered terminal differentiation by suppressing the expression of key adipogenic regulators, C/EBP-α and PPAR-γ (Munekata and Sakamoto 2009). These findings align with data obtained from human adipose-derived stem cells, where FOXO1 plays a positive role by maintaining cellular redox balance and promoting adipogenic differentiation (Higuchi et al. 2013).

However, contrasting results were reported by Nakae et al., who introduced a constitutively active mutant FOXO1 in mouse 3T3-F442A preadipocytes. They argue that FOXO1 exerts an inhibitory effect on adipogenesis through a multifaceted mechanism (Nakae et al. 2003). FOXO1 induces early growth arrest by increasing the expression of cell cycle inhibitors such as p21 and p27, which can impede the expansion of committed preadipocytes. Additionally, FOXO1 may directly inhibit C/EBP-dependent terminal differentiation by enhancing CHOP10 expression (Nakae et al. 2003). These diverse findings suggest that the role of FOXO1 in adipogenic differentiation is influenced by the specific cell type and the cellular context. Further research is needed to fully understand the complex regulatory mechanisms underlying adipogenesis and the precise role of FOXO1 in this process.

#### Hematopoietic differentiation

Hematopoietic differentiation is a complex and dynamic process that ensures the production of a diverse range of blood cells essential for immune function and blood homeostasis (Pinho and Frenette 2019; Wilson and Trumpp 2006). One crucial step in this process is lineage commitment, where HSCs decide to differentiate into either the myeloid or lymphoid lineage. FOXO transcription factors play a significant role in orchestrating this intricate process, ensuring the proper development and differentiation of HSCs into their respective lymphoid or myeloid cell lineages.

FOXO1 is required for lymphoid lineage commitment and differentiation (Dengler et al. 2008; Mansson et al. 2012). Early deletion of FOXO1 in nascent pro-B cells leads to a block in B cell development, primarily due to impaired expression of interleukin 7 receptor α (IL-7Rα). Inactivating FOXO1 in late pro-B cells leads to an arrest at the pre-B cell stage, primarily due to reduced expression of RAG1 and RAG2 (Dengler et al. 2008). Additionally, FOXO1 plays a role in specifying B-cell fate by upregulating the expression of early B-cell factor 1 (EBF1), which, in turn, activates FOXO1 expression through a positive feedback loop, enhancing and stabilizing B-cell fate (Mansson et al. 2012).

**Fig. 3:**
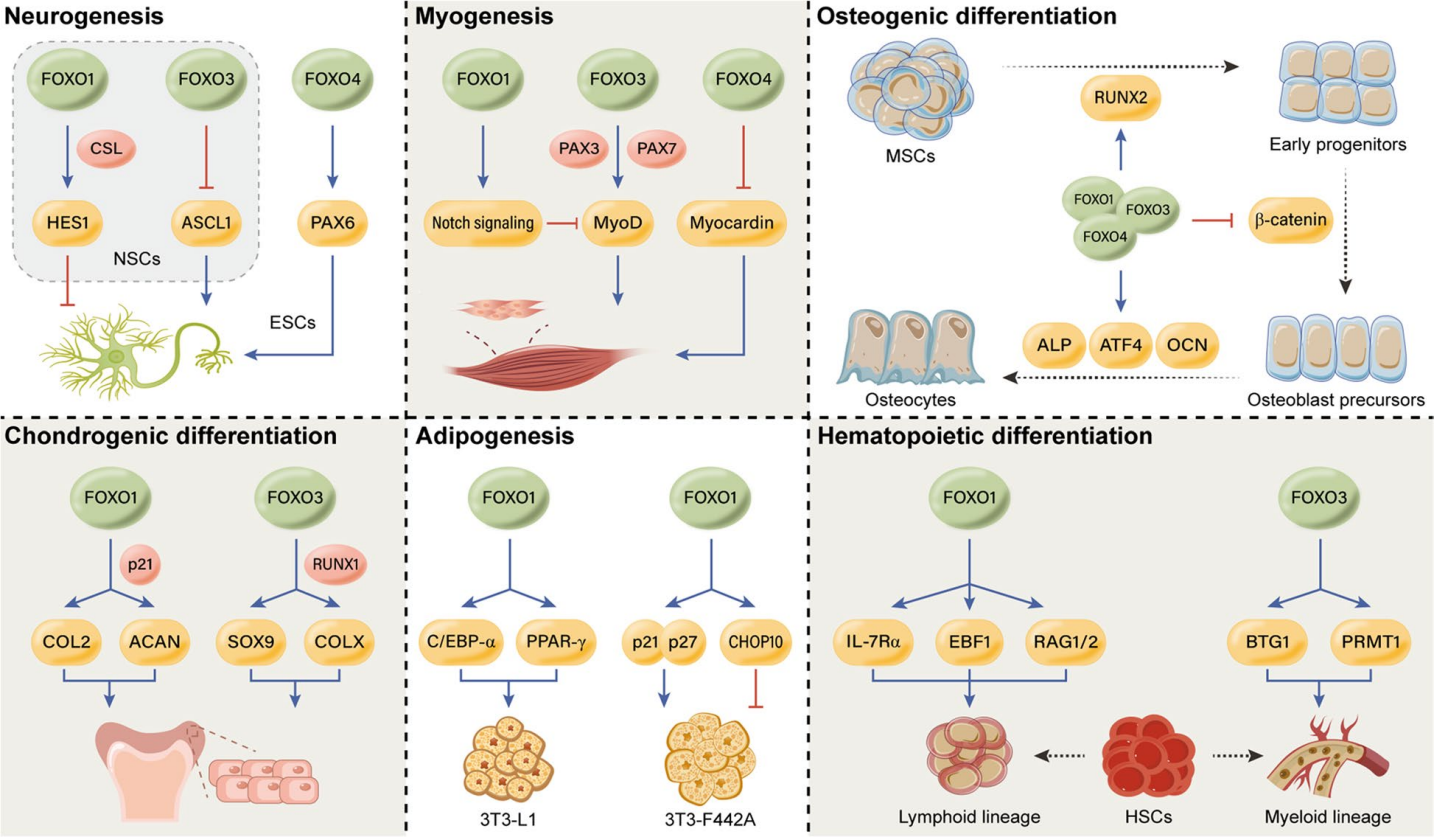
Context-dependent regulation of FOXOs in stem cell differentiation. FOXO transcription factors play a pivotal role in stem cell differentiation by orchestrating gene expression and cellular processes. However, their impact on differentiation is nuanced and contingent upon specific contexts, with the potential for both promotion and inhibition based on their transcriptional targets. Different members of the FOXO family exhibit distinct roles in stem cell differentiation; for example, FOXO1/3 inhibits neural stem cell (NSC) differentiation into neurons, while FOXO4 promotes neurogenesis of embryonic stem cells (ESCs). FOXO1/4 inhibits myoblast differentiation, whereas FOXO3 promotes the differentiation of satellite cells. During osteogenic differentiation, FOXOs exert stage-specific functions through interactions with various factors, such as RUNX2 in early progenitors, β‐catenin in osteoblast precursors, and ALP, ATF4, and OCN in mature osteoblasts. Both FOXO1 and FOXO3 are essential for mesenchymal stem cells (MSCs) to differentiate into chondrocytes. The role of FOXO1 in adipogenic differentiation is influenced by the specific cell type and cellular context. Furthermore, FOXO1 and FOXO3 are indispensable for the proper development and differentiation of hematopoietic stem cells (HSCs) into their respective lymphoid or myeloid cell lineages

On the other hand, FOXO3 is necessary for myeloid cell differentiation (Bakker et al. 2004; Kang et al. 2015). During erythroid differentiation, there is an increase in both the expression and nuclear accumulation of FOXO3. Premature activation of FOXO3 leads to accelerated differentiation of erythroid progenitors into mature erythrocytes. This process relies on the upregulation of B cell translocation gene 1 (BTG1) and subsequent activation of protein arginine methyltransferase 1 (PRMT1) (Bakker et al. 2004). FOXO3 also plays a critical role in erythropoiesis by maintaining low levels of reactive oxygen species (ROS), which increase erythrocyte lifespan and maturation (Marinkovic et al. 2007). While excessive ROS can be detrimental to cells, a moderate and developmentally regulated level of ROS can be beneficial. This has been observed in Drosophila multipotent hematopoietic progenitors, which share functional similarities with mammalian myeloid progenitors. Activation of FOXO through high levels of ROS in these progenitors promotes their differentiation into mature blood cells (Evans et al. 2003; Owusu-Ansah and Banerjee 2009). Taken together, these findings highlight the intricate and context-dependent role of FOXOs in the differentiation of stem cells (Fig. 3).

## Conclusions and perspectives

**Fig. 4:**
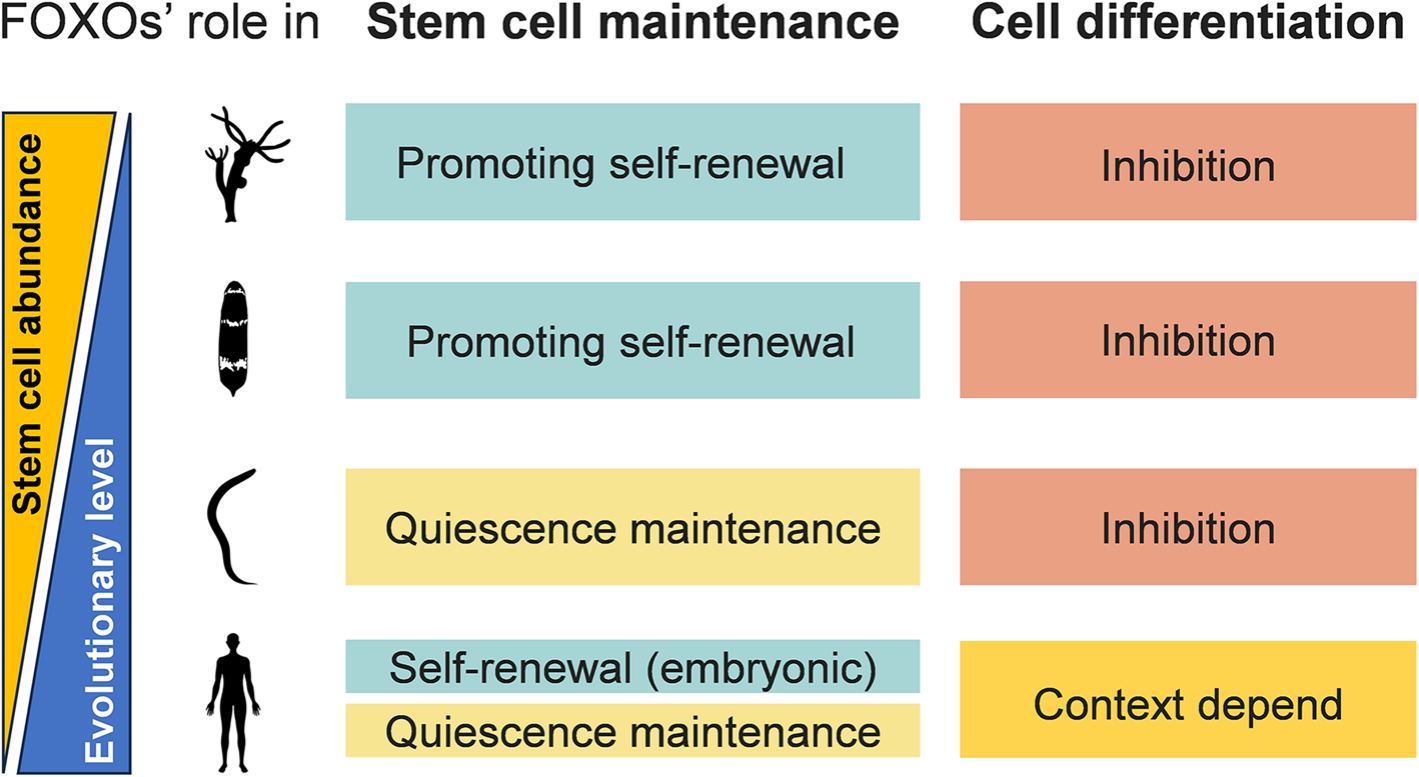
Evolutionary role of FOXOs in the regulation of stem cell fate. In organisms with abundant pluripotent stem cells like cnidarians and acoels, FOXOs promote stem cell self-renewal, enabling rapid regeneration of body parts after injury. This strategy is also observed in mammalian embryonic stem cells (ESCs). However, FOXOs in adult mammals primarily regulate quiescence in species with limited adult stem cells (ASCs), preserving the stem cell pool and minimizing depletion or inappropriate cell growth. FOXOs also play a role in stem cell differentiation, either promoting or inhibiting it as needed for tissue homeostasis. This functional divergence of FOXO genes allows organisms to balance tissue maintenance and long-term preservation of the stem cell pool

Understanding the mechanisms that govern stem cell fate is crucial for unraveling the complexities of development, tissue homeostasis, and regenerative potential. Increasing evidence supports the essential roles of FOXOs in stem cell fate decisions (Liang and Ghaffari 2017; Ludikhuize and Rodriguez Colman 2021; Ro et al. 2013). In the current review, we summarize the structure and regulation of FOXO proteins and their roles in steering the fate of stem cells. On the one hand, FOXOs play a conserved role in maintaining stem cells, either by promoting their self-renewal or maintaining quiescence. Interestingly, their strategies for mobilizing stem cells vary in organisms at different evolutionary levels. In lower animals with abundant pluripotent stem cells, such as cnidarians and acoels, FOXOs promote stem cell self-renewal constitutively (Boehm et al. 2012; Kimura et al. 2022). This allows the rapid thriving of stem cells in response to injury, facilitating the efficient regeneration of whole-body parts and providing a survival advantage in fluctuating environments. However, in invertebrates with limited pluripotent ASCs, such as *C. elegans*, FOXO-mediated cell renewal has evolved to perform other tasks related to quiescence maintenance, preventing premature exhaust (Baugh and Sternberg 2006; Karp and Greenwald 2013; Lamitina and Strange 2005). In higher species like mammals, both mechanisms are present, but they operate at different developmental stages. FOXO1 upregulates pluripotent genes to maintain hESCs in a state of self-renewal, ensuring a constant supply of developmental potential (Zhang et al. 2011b). However, as ESCs gradually lose their pluripotency during embryonic development, FOXOs play a role in maintaining a quiescent state of ASCs and tightly regulate their activation (Schaible and Sussman 2013). This quiescent state preserves the stem cell pool and minimizes the risk of depletion or inappropriate cell growth. On the other hand, FOXOs generally prevent stem cell differentiation to preserve their pool among species. However, as organisms with greater organizational, behavioral, and life-history complexity evolved, FOXOs developed a more flexible role in stem cell differentiation, capable of either promoting or inhibiting cell differentiation in a context-dependent manner (Kim et al. 2015; Ludikhuize and Rodriguez Colman 2021; Vilchez et al. 2013). While inhibiting stem cell differentiation is crucial for maintaining the stem cell pool, promoting it helps replenish the pool of specialized cells required for tissue homeostasis. This adaptability likely emerged with the increasing complexity of organisms, leading to tissue-specific adaptations and a shift towards maintaining tissue homeostasis over a longer lifespan in higher animals such as mammals. This enables the regulation of stem cell behavior to ensure proper tissue maintenance and repair. To this end, the functional divergence of FOXO genes in the regulation of stem cell behaviors enables organisms to strike a delicate balance between tissue homeostasis and the long-term preservation of the stem cell pool (Fig. 4).

Gene duplication is considered to be a crucial factor in driving functional divergence (Lynch et al. 2006). The abundance of FOXO isoforms in higher animals supports the molecular basis for diversifying their functions. Phylogenetic analysis as that conducted by Wang et al. has shown that vertebrate FOXO genes originated from successive gene duplications, the first leading to the emergence of FOXO3/6 and FOXO1/4 lineages, followed by two additional duplications resulting in the four current genes (Wang et al. 2009). When a gene is duplicated, one of the paralogs can evolve and diversify towards a new functionality, while the other paralog retains its original function. FOXOs generated by gene duplications have conserved structural domains across species. Comparing human FOXOs with their orthologs in invertebrates such as *C. elegans* and *Drosophila*, FOXO3 shares the most amino acid identity (Schmitt-Ney 2020), while FOXO6 evolved at a faster rate compared to its counterparts (Wang et al. 2009). Previous research has reported that human FOXO3 can partially substitute DAF-16 in *C. elegans*, demonstrating the functional compatibility of FOXOs in different species (Lee et al. 2001). However, gene knockout studies targeting mammalian FOXOs have uncovered unique roles for this subfamily in development (Castrillon et al. 2003; Hosaka et al. 2004). For instance, FOXO1 knockout mice experience embryonic lethality due to vascular development issues, while FOXO3 knockout mice survive to adulthood but suffer from abnormal ovarian follicular development, resulting in infertility. FOXO4 knockout mice, on the other hand, fail to reveal any obvious phenotypic abnormalities, indicating the presence of compensatory functions by other FOXOs. Lastly, FOXO6 deficiency leads to decreased dendritic spine density in hippocampal neurons and impaired synaptic function (Salih et al. 2012). The conserved structures of FOXO proteins while evolving divergent functions raise intriguing questions. This can be attributed to their distinct expression patterns, post-translational modifications, and interacting partners.

First, different FOXO members exhibit unique expression patterns. For instance, human FOXO genes located on different chromosomes show autonomous expression influenced by their chromatin environments, gene promoters, and enhancers (Link 2019). Additionally, they display distinct tissue distribution patterns. FOXO1 is mainly found in adipose tissue, while FOXO3 is more abundant in cardiac and skeletal muscles. FOXO4, on the other hand, is mainly expressed in the heart, brain, and spleen (Furuyama et al. 2000). FOXO6 is dominantly present in the developing and adult brain (Jacobs et al. 2003), aligning with its significant role in memory consolidation (Salih et al. 2012). These tissue-specific distributions of FOXO proteins enable them to respond to distinct signaling cues and exert diverse functions.

Second, FOXO activity can be regulated at multiple levels. FOXOs undergo various PTMs such as phosphorylation, acetylation, ubiquitination, methylation, and GlcNAcylation (Calissi et al. 2021; Hu et al. 2019; Rodriguez-Colman et al. 2023). These modifications can either activate or inhibit FOXO activity, depending on the specific modification and cellular context. Several miRNAs have also been described to fine-tune FOXO mRNA stability (Urbanek and Klotz 2017). RNA modification, such as m6A methylation, and RNA-binding proteins also influence FOXO activity at the post-transcriptional level (Guo et al. 2014; Li et al. 2013; Yu et al. 2014). The dynamic regulation of FOXO proteins at multiple levels ensures their precise control over gene expression, enabling them to carry out specific functions in a context-dependent manner.

Third, FOXOs interact with various co-regulators to enhance their binding to specific target promoters. For example, the interaction between CBP/p300 and FOXOs boosts the transcription of genes related to insulin signaling, such as the IGF-binding protein-1 (Perrot and Rechler 2005). The interaction of DYRK1 with FOXO1 enhances the transcription of the target gene G6P, which is involved in gluconeogenesis (von Groote-Bidlingmaier et al. 2003). On the contrary, SIN3A acts as a corepressor of FOXO1 and inhibits the expression of glucokinase (Langlet et al. 2017). Notably, FOXOs can also exert transcription-independent functions through their interaction with co-regulators. For instance, cytosolic FOXO1 interacts with ATG7 to elicit autophagy in response to stress (Zhao et al. 2010), while FOXO3 recruits p53 to the cytoplasm, promoting apoptosis (You et al. 2006). FOXO3 also interacts with ATM, a serine/threonine kinase, to regulate DNA damage responses by activating downstream mediators like H2AX (Tsai et al. 2008). Overall, these interactions of FOXOs with co-regulators contribute to the diverse functions of FOXOs in a wide range of cellular functions.

In summary, the exact roles of FOXOs in regulating stem cell fate are complicated and vary in a context-dependent manner. Therefore, understanding the precise mechanisms by which FOXOs regulate stem cell fate determination holds great promise for therapeutic applications. It can lead to the development of strategies to manipulate stem cell fate, optimize protocols for generating specific cell types, and improve stem cell-based regenerative therapies. Additionally, FOXOs have been linked to the control of aging and lifespan (Lin et al. 1997; Martins et al. 2016; Morris et al. 2015). Additional investigation into their influences on determining stem cell fate can shed light on the mechanisms underlying age-related decline in stem cell function. This understanding could aid in the development of interventions aimed at rejuvenating aged stem cells, potentially delaying or reversing age-related degenerative processes. Furthermore, pharmaceutical targeting FOXO functions may present new therapeutic approaches for diseases such as cancer (Calissi et al. 2021; Farhan et al. 2017; Orea-Soufi et al. 2022). Overall, further exploration of FOXO’s role in stem cell fate holds great promise for advancing stem cell biology and its therapeutic uses.

## Data Availability

Not applicable.

## Abbreviations

FOXO: Forkhead box O
DBD: DNA-binding domain
TAD: Transcriptional activation domain
PTM: Post-translational modification
miRNA: MicroRNA
MSCs: Mesenchymal stem cells
ESCs: Embryonic stem cells
ASCs: Adult stem cells
SCs: Satellite cells
NSCs: Neural stem cells
HSCs: Hematopoietic stem cells

## References

[CR1] Akan I, Halim A, Vakhrushev SY, Clausen H, Hanover JA (2021). Drosophila O-GlcNAcase mutants reveal an expanded glycoproteome and novel growth and longevity phenotypes. Cells.

[CR2] Almeida M, Han L, Martin-Millan M, O’Brien CA, Manolagas SC (2007). Oxidative stress antagonizes Wnt signaling in osteoblast precursors by diverting beta-catenin from T cell factor- to forkhead box O-mediated transcription. J Biol Chem.

[CR3] Ambrogini E, Almeida M, Martin-Millan M, Paik JH, Depinho RA, Han L (2010). FoxO-mediated defense against oxidative stress in osteoblasts is indispensable for skeletal homeostasis in mice. Cell Metab.

[CR4] Anderson MJ, Viars CS, Czekay S, Cavenee WK, Arden KC (1998). Cloning and characterization of three human forkhead genes that comprise an FKHR-like gene subfamily. Genomics.

[CR5] Arai F, Hirao A, Ohmura M, Sato H, Matsuoka S, Takubo K (2004). Tie2/angiopoietin-1 signaling regulates hematopoietic stem cell quiescence in the bone marrow niche. Cell.

[CR6] Artavanis-Tsakonas S, Rand MD, Lake RJ (1999). Notch signaling: cell fate control and signal integration in development. Science.

[CR7] Audesse AJ, Dhakal S, Hassell LA, Gardell Z, Nemtsova Y, Webb AE (2019). FOXO3 directly regulates an autophagy network to functionally regulate proteostasis in adult neural stem cells. PLoS Genet.

[CR8] Bakker WJ, Blazquez-Domingo M, Kolbus A, Besooyen J, Steinlein P, Beug H (2004). FoxO3a regulates erythroid differentiation and induces BTG1, an activator of protein arginine methyl transferase 1. J Cell Biol.

[CR9] Barker N, Bartfeld S, Clevers H (2010). Tissue-resident adult stem cell populations of rapidly self-renewing organs. Cell Stem Cell.

[CR10] Bartel DP (2009). MicroRNAs: target recognition and regulatory functions. Cell.

[CR11] Baugh LR, Sternberg PW (2006). DAF-16/FOXO regulates transcription of cki-1/Cip/Kip and repression of lin-4 during C. elegans L1 arrest. Curr Biol.

[CR12] Becher J, Simula L, Volpe E, Procaccini C, La Rocca C, D’Acunzo P (2018). AMBRA1 controls regulatory T-cell differentiation and homeostasis upstream of the FOXO3-FOXP3 axis. Dev Cell.

[CR13] Bell DM, Leung KK, Wheatley SC, Ng LJ, Zhou S, Ling KW (1997). SOX9 directly regulates the type-II collagen gene. Nat Genet.

[CR14] Biggs WH, Meisenhelder J, Hunter T, Cavenee WK, Arden KC (1999). Protein kinase B/Akt-mediated phosphorylation promotes nuclear exclusion of the winged helix transcription factor FKHR1. Proc Natl Acad Sci U S A.

[CR15] Bjornson CR, Cheung TH, Liu L, Tripathi PV, Steeper KM, Rando TA (2012). Notch signaling is necessary to maintain quiescence in adult muscle stem cells. Stem Cells.

[CR16] Boehm AM, Khalturin K, Anton-Erxleben F, Hemmrich G, Klostermeier UC, Lopez-Quintero JA (2012). FoxO is a critical regulator of stem cell maintenance in immortal Hydra. Proc Natl Acad Sci U S A.

[CR17] Boo SH, Kim YK (2020). The emerging role of RNA modifications in the regulation of mRNA stability. Exp Mol Med.

[CR18] Borkhardt A, Repp R, Haas OA, Leis T, Harbott J, Kreuder J (1997). Cloning and characterization of AFX, the gene that fuses to MLL in acute leukemias with a t(X;11)(q13;q23). Oncogene.

[CR19] Bosch TC (2009). Hydra and the evolution of stem cells. BioEssays.

[CR20] Boulias K, Horvitz HR (2012). The C. elegans microRNA mir-71 acts in neurons to promote germline-mediated longevity through regulation of DAF-16/FOXO. Cell Metab.

[CR21] Boyer LA, Lee TI, Cole MF, Johnstone SE, Levine SS, Zucker JP (2005). Core transcriptional regulatory circuitry in human embryonic stem cells. Cell.

[CR22] Brenkman AB, de Keizer PL, van den Broek NJ, Jochemsen AG, Burgering BM (2008). Mdm2 induces mono-ubiquitination of FOXO4. PLoS One.

[CR23] Bridge D, Theofiles AG, Holler RL, Marcinkevicius E, Steele RE, Martinez DE (2010). FoxO and stress responses in the cnidarian Hydra vulgaris. PLoS One.

[CR24] Brown AK, Webb AE (2018). Regulation of FOXO factors in mammalian cells. Curr Top Dev Biol.

[CR25] Brownawell AM, Kops GJ, Macara IG, Burgering BM (2001). Inhibition of nuclear import by protein kinase B (Akt) regulates the subcellular distribution and activity of the forkhead transcription factor AFX. Mol Cell Biol.

[CR26] Brunet A, Bonni A, Zigmond MJ, Lin MZ, Juo P, Hu LS (1999). Akt promotes cell survival by phosphorylating and inhibiting a Forkhead transcription factor. Cell.

[CR27] Brunet A, Park J, Tran H, Hu LS, Hemmings BA, Greenberg ME (2001). Protein kinase SGK mediates survival signals by phosphorylating the forkhead transcription factor FKHRL1 (FOXO3a). Mol Cell Biol.

[CR28] Brunet A, Kanai F, Stehn J, Xu J, Sarbassova D, Frangioni JV (2002). 14-3-3 transits to the nucleus and participates in dynamic nucleocytoplasmic transport. J Cell Biol.

[CR29] Brunet A, Sweeney LB, Sturgill JF, Chua KF, Greer PL, Lin Y (2004). Stress-dependent regulation of FOXO transcription factors by the SIRT1 deacetylase. Science.

[CR30] Buckley SM, Aranda-Orgilles B, Strikoudis A, Apostolou E, Loizou E, Moran-Crusio K (2012). Regulation of pluripotency and cellular reprogramming by the ubiquitin-proteasome system. Cell Stem Cell.

[CR31] Cahill CM, Tzivion G, Nasrin N, Ogg S, Dore J, Ruvkun G (2001). Phosphatidylinositol 3-kinase signaling inhibits DAF-16 DNA binding and function via 14-3-3-dependent and 14-3-3-independent pathways. J Biol Chem.

[CR32] Calissi G, Lam EW, Link W (2021). Therapeutic strategies targeting FOXO transcription factors. Nat Rev Drug Discov.

[CR33] Calnan DR, Webb AE, White JL, Stowe TR, Goswami T, Shi X (2012). Methylation by Set9 modulates FoxO3 stability and transcriptional activity. Aging (Albany NY).

[CR34] Castrillon DH, Miao L, Kollipara R, Horner JW, DePinho RA (2003). Suppression of ovarian follicle activation in mice by the transcription factor Foxo3a. Science.

[CR35] Castro DS, Skowronska-Krawczyk D, Armant O, Donaldson IJ, Parras C, Hunt C (2006). Proneural bHLH and Brn proteins coregulate a neurogenic program through cooperative binding to a conserved DNA motif. Dev Cell.

[CR36] Chakkalakal JV, Jones KM, Basson MA, Brack AS (2012). The aged niche disrupts muscle stem cell quiescence. Nature.

[CR37] Chapuis N, Park S, Leotoing L, Tamburini J, Verdier F, Bardet V (2010). IkappaB kinase overcomes PI3K/Akt and ERK/MAPK to control FOXO3a activity in acute myeloid leukemia. Blood.

[CR38] Charge SB, Rudnicki MA (2004). Cellular and molecular regulation of muscle regeneration. Physiol Rev.

[CR39] Chen AT, Guo C, Dumas KJ, Ashrafi K, Hu PJ (2013). Effects of Caenorhabditis elegans sgk-1 mutations on lifespan, stress resistance, and DAF-16/FoxO regulation. Aging Cell.

[CR40] Chen B, Bao Y, Chen X, Yi J, Liu S, Fang Z (2015). Mir-664 promotes osteosarcoma cells proliferation via downregulating of FOXO4. Biomed Pharmacother.

[CR41] Chen D, Gong Y, Xu L, Zhou M, Li J, Song J (2019). Bidirectional regulation of osteogenic differentiation by the FOXO subfamily of Forkhead transcription factors in mammalian MSCs. Cell Prolif.

[CR42] Chen K, Gao P, Li Z, Dai A, Yang M, Chen S (2022). Forkhead box O signaling pathway in skeletal muscle atrophy. Am J Pathol.

[CR43] Choi S, Jeong HJ, Kim H, Choi D, Cho SC, Seong JK (2019). Skeletal muscle-specific Prmt1 deletion causes muscle atrophy via deregulation of the PRMT6-FOXO3 axis. Autophagy.

[CR44] Coolican SA, Samuel DS, Ewton DZ, McWade FJ, Florini JR (1997). The mitogenic and myogenic actions of insulin-like growth factors utilize distinct signaling pathways. J Biol Chem.

[CR45] Cristancho AG, Lazar MA (2011). Forming functional fat: a growing understanding of adipocyte differentiation. Nat Rev Mol Cell Biol.

[CR46] Daitoku H, Hatta M, Matsuzaki H, Aratani S, Ohshima T, Miyagishi M (2004). Silent information regulator 2 potentiates Foxo1-mediated transcription through its deacetylase activity. Proc Natl Acad Sci U S A.

[CR47] Dansen TB, Burgering BM (2008). Unravelling the tumor-suppressive functions of FOXO proteins. Trends Cell Biol.

[CR48] Dansen TB, Smits LM, van Triest MH, de Keizer PL, van Leenen D, Koerkamp MG (2009). Redox-sensitive cysteines bridge p300/CBP-mediated acetylation and FoxO4 activity. Nat Chem Biol.

[CR49] de Morree A, Rando TA (2023). Regulation of adult stem cell quiescence and its functions in the maintenance of tissue integrity. Nat Rev Mol Cell Biol.

[CR50] De Ruiter ND, Burgering BM, Bos JL (2001). Regulation of the Forkhead transcription factor AFX by Ral-dependent phosphorylation of threonines 447 and 451. Mol Cell Biol.

[CR51] Dengler HS, Baracho GV, Omori SA, Bruckner S, Arden KC, Castrillon DH (2008). Distinct functions for the transcription factor Foxo1 at various stages of B cell differentiation. Nat Immunol.

[CR52] Ducy P, Desbois C, Boyce B, Pinero G, Story B, Dunstan C (1996). Increased bone formation in osteocalcin-deficient mice. Nature.

[CR53] Duwe L, Munoz-Garrido P, Lewinska M, Lafuente-Barquero J, Satriano L, Hogdall D (2023). MicroRNA-27a-3p targets FoxO signalling to induce tumour-like phenotypes in bile duct cells. J Hepatol.

[CR54] Eijkelenboom A, Burgering BM (2013). FOXOs: signalling integrators for homeostasis maintenance. Nat Rev Mol Cell Biol.

[CR55] Essers MA, Weijzen S, de Vries-Smits AM, Saarloos I, de Ruiter ND, Bos JL (2004). FOXO transcription factor activation by oxidative stress mediated by the small GTPase Ral and JNK. EMBO J.

[CR56] Evans CJ, Hartenstein V, Banerjee U (2003). Thicker than blood: conserved mechanisms in Drosophila and vertebrate hematopoiesis. Dev Cell.

[CR57] Farhan M, Wang H, Gaur U, Little PJ, Xu J, Zheng W (2017). FOXO signaling pathways as therapeutic targets in cancer. Int J Biol Sci.

[CR58] Fei M, Zhao Y, Wang Y, Lu M, Cheng C, Huang X (2009). Low expression of Foxo3a is associated with poor prognosis in ovarian cancer patients. Cancer Invest.

[CR59] Fielenbach N, Antebi AC (2008). elegans dauer formation and the molecular basis of plasticity. Genes Dev.

[CR60] Fuentealba LC, Obernier K, Alvarez-Buylla A (2012). Adult neural stem cells bridge their niche. Cell Stem Cell.

[CR61] Fukuoka M, Daitoku H, Hatta M, Matsuzaki H, Umemura S, Fukamizu A (2003). Negative regulation of forkhead transcription factor AFX (Foxo4) by CBP-induced acetylation. Int J Mol Med.

[CR62] Furutachi S, Miya H, Watanabe T, Kawai H, Yamasaki N, Harada Y (2015). Slowly dividing neural progenitors are an embryonic origin of adult neural stem cells. Nat Neurosci.

[CR63] Furuyama T, Nakazawa T, Nakano I, Mori N (2000). Identification of the differential distribution patterns of mRNAs and consensus binding sequences for mouse DAF-16 homologues. Biochem J.

[CR64] Garcia-Prat L, Perdiguero E, Alonso-Martin S, Dell’Orso S, Ravichandran S, Brooks SR (2020). FoxO maintains a genuine muscle stem-cell quiescent state until geriatric age. Nat Cell Biol.

[CR65] Gehrke AR, Neverett E, Luo YJ, Brandt A, Ricci L, Hulett RE (2019). Acoel genome reveals the regulatory landscape of whole-body regeneration. Science.

[CR66] Glass DA, Bialek P, Ahn JD, Starbuck M, Patel MS, Clevers H (2005). Canonical Wnt signaling in differentiated osteoblasts controls osteoclast differentiation. Dev Cell.

[CR67] Gopinath SD, Webb AE, Brunet A, Rando TA (2014). FOXO3 promotes quiescence in adult muscle stem cells during the process of self-renewal. Stem Cell Reports.

[CR68] Greer EL, Oskoui PR, Banko MR, Maniar JM, Gygi MP, Gygi SP (2007). The energy sensor AMP-activated protein kinase directly regulates the mammalian FOXO3 transcription factor. J Biol Chem.

[CR69] Gregorian C, Nakashima J, Le Belle J, Ohab J, Kim R, Liu A (2009). Pten deletion in adult neural stem/progenitor cells enhances constitutive neurogenesis. J Neurosci.

[CR70] Guo S, Rena G, Cichy S, He X, Cohen P, Unterman T (1999). Phosphorylation of serine 256 by protein kinase B disrupts transactivation by FKHR and mediates effects of insulin on insulin-like growth factor-binding protein-1 promoter activity through a conserved insulin response sequence. J Biol Chem.

[CR71] Guo W, Jiang T, Lian C, Wang H, Zheng Q, Ma H (2014). QKI deficiency promotes FoxO1 mediated nitrosative stress and endoplasmic reticulum stress contributing to increased vulnerability to ischemic injury in diabetic heart. J Mol Cell Cardiol.

[CR72] Habashy HO, Rakha EA, Aleskandarany M, Ahmed MA, Green AR, Ellis IO (2011). FOXO3a nuclear localisation is associated with good prognosis in luminal-like breast cancer. Breast Cancer Res Treat.

[CR73] Hart GW, Housley MP, Slawson C (2007). Cycling of O-linked beta-N-acetylglucosamine on nucleocytoplasmic proteins. Nature.

[CR74] Hemmrich G, Khalturin K, Boehm AM, Puchert M, Anton-Erxleben F, Wittlieb J (2012). Molecular signatures of the three stem cell lineages in hydra and the emergence of stem cell function at the base of multicellularity. Mol Biol Evol.

[CR75] Hennessy BT, Smith DL, Ram PT, Lu Y, Mills GB (2005). Exploiting the PI3K/AKT pathway for cancer drug discovery. Nat Rev Drug Discov.

[CR76] Hertweck M, Gobel C, Baumeister RC (2004). elegans SGK-1 is the critical component in the Akt/PKB kinase complex to control stress response and life span. Dev Cell.

[CR77] Higuchi M, Dusting GJ, Peshavariya H, Jiang F, Hsiao ST, Chan EC (2013). Differentiation of human adipose-derived stem cells into fat involves reactive oxygen species and Forkhead box O1 mediated upregulation of antioxidant enzymes. Stem Cells Dev.

[CR78] Hitoshi S, Alexson T, Tropepe V, Donoviel D, Elia AJ, Nye JS (2002). Notch pathway molecules are essential for the maintenance, but not the generation, of mammalian neural stem cells. Genes Dev.

[CR79] Ho SR, Wang K, Whisenhunt TR, Huang P, Zhu X, Kudlow JE (2010). O-GlcNAcylation enhances FOXO4 transcriptional regulation in response to stress. FEBS Lett.

[CR80] Ho KK, McGuire VA, Koo CY, Muir KW, de Olano N, Maifoshie E (2012). Phosphorylation of FOXO3a on Ser-7 by p38 promotes its nuclear localization in response to doxorubicin. J Biol Chem.

[CR81] Hornsveld M, Dansen TB, Derksen PW, Burgering BMT (2018). Re-evaluating the role of FOXOs in cancer. Semin Cancer Biol.

[CR82] Hosaka T, Biggs WH, Tieu D, Boyer AD, Varki NM, Cavenee WK (2004). Disruption of forkhead transcription factor (FOXO) family members in mice reveals their functional diversification. Proc Natl Acad Sci U S A.

[CR83] Housley MP, Rodgers JT, Udeshi ND, Kelly TJ, Shabanowitz J, Hunt DF (2008). O-GlcNAc regulates FoxO activation in response to glucose. J Biol Chem.

[CR84] Housley MP, Udeshi ND, Rodgers JT, Shabanowitz J, Puigserver P, Hunt DF (2009). A PGC-1alpha-O-GlcNAc transferase complex regulates FoxO transcription factor activity in response to glucose. J Biol Chem.

[CR85] Hribal ML, Nakae J, Kitamura T, Shutter JR, Accili D (2003). Regulation of insulin-like growth factor-dependent myoblast differentiation by Foxo forkhead transcription factors. J Cell Biol.

[CR86] Hu MC, Lee DF, Xia W, Golfman LS, Ou-Yang F, Yang JY (2004). IkappaB kinase promotes tumorigenesis through inhibition of forkhead FOXO3a. Cell.

[CR87] Hu H, Hilton MJ, Tu X, Yu K, Ornitz DM, Long F (2005). Sequential roles of Hedgehog and Wnt signaling in osteoblast development. Development.

[CR88] Hu P, Geles KG, Paik JH, DePinho RA, Tjian R (2008). Codependent activators direct myoblast-specific MyoD transcription. Dev Cell.

[CR89] Hu T, Chung YM, Guan M, Ma M, Ma J, Berek JS (2014). Reprogramming ovarian and breast cancer cells into non-cancerous cells by low-dose metformin or SN-38 through FOXO3 activation. Sci Rep.

[CR90] Hu W, Yang Z, Yang W, Han M, Xu B, Yu Z (2019). Roles of forkhead box O (FoxO) transcription factors in neurodegenerative diseases: a panoramic view. Prog Neurobiol.

[CR91] Huang H, Tindall DJ (2007). Dynamic FoxO transcription factors. J Cell Sci.

[CR92] Huang H, Regan KM, Wang F, Wang D, Smith DI, van Deursen JM (2005). Skp2 inhibits FOXO1 in tumor suppression through ubiquitin-mediated degradation. Proc Natl Acad Sci U S A.

[CR93] Huang H, Regan KM, Lou Z, Chen J, Tindall DJ (2006). CDK2-dependent phosphorylation of FOXO1 as an apoptotic response to DNA damage. Science.

[CR94] Ikegami D, Akiyama H, Suzuki A, Nakamura T, Nakano T, Yoshikawa H (2011). Sox9 sustains chondrocyte survival and hypertrophy in part through Pik3ca-Akt pathways. Development.

[CR95] Ishibashi M, Moriyoshi K, Sasai Y, Shiota K, Nakanishi S, Kageyama R (1994). Persistent expression of helix-loop-helix factor HES-1 prevents mammalian neural differentiation in the central nervous system. EMBO J.

[CR96] Isik M, Blackwell TK, Berezikov E (2016). MicroRNA mir-34 provides robustness to environmental stress response via the DAF-16 network in C. elegans. Sci Rep.

[CR97] Iyer S, Ambrogini E, Bartell SM, Han L, Roberson PK, de Cabo R (2013). FOXOs attenuate bone formation by suppressing Wnt signaling. J Clin Invest.

[CR98] Jacobs FM, van der Heide LP, Wijchers PJ, Burbach JP, Hoekman MF, Smidt MP (2003). FoxO6, a novel member of the FoxO class of transcription factors with distinct shuttling dynamics. J Biol Chem.

[CR99] Jiang BH, Aoki M, Zheng JZ, Li J, Vogt PK (1999). Myogenic signaling of phosphatidylinositol 3-kinase requires the serine-threonine kinase Akt/protein kinase B. Proc Natl Acad Sci U S A.

[CR100] Jiang Y, Zou L, Lu WQ, Zhang Y, Shen AG (2013). Foxo3a expression is a prognostic marker in breast cancer. PLoS One.

[CR101] Jones KT, Greer ER, Pearce D, Ashrafi K (2009). Rictor/TORC2 regulates Caenorhabditis elegans fat storage, body size, and development through sgk-1. PLoS Biol.

[CR102] Kageyama R, Ohtsuka T (1999). The Notch-Hes pathway in mammalian neural development. Cell Res.

[CR103] Kang H, Corr M, Mansson R, Welinder E, Hedrick SM, Stone EL (2015). Loss of murine FOXO3 in cells of the myeloid lineage enhances myelopoiesis but protects from K/BxN-serum transfer-induced arthritis. PLoS One.

[CR104] Karp X, Greenwald I (2013). Control of cell-fate plasticity and maintenance of multipotency by DAF-16/FoxO in quiescent Caenorhabditis elegans. Proc Natl Acad Sci U S A.

[CR105] Kashii Y, Uchida M, Kirito K, Tanaka M, Nishijima K, Toshima M (2000). A member of Forkhead family transcription factor, FKHRL1, is one of the downstream molecules of phosphatidylinositol 3-kinase-Akt activation pathway in erythropoietin signal transduction. Blood.

[CR106] Kato S, Ding J, Pisck E, Jhala US, Du K (2008). COP1 functions as a FoxO1 ubiquitin E3 ligase to regulate FoxO1-mediated gene expression. J Biol Chem.

[CR107] Kenny TC, Hart P, Ragazzi M, Sersinghe M, Chipuk J, Sagar MAK (2017). Selected mitochondrial DNA landscapes activate the SIRT3 axis of the UPR(mt) to promote metastasis. Oncogene.

[CR108] Kenyon C, Chang J, Gensch E, Rudner A, Tabtiang RAC (1993). elegans mutant that lives twice as long as wild type. Nature.

[CR109] Khongkow M, Olmos Y, Gong C, Gomes AR, Monteiro LJ, Yague E (2013). SIRT6 modulates paclitaxel and epirubicin resistance and survival in breast cancer. Carcinogenesis.

[CR110] Kim DH, Perdomo G, Zhang T, Slusher S, Lee S, Phillips BE (2011). FoxO6 integrates insulin signaling with gluconeogenesis in the liver. Diabetes.

[CR111] Kim KM, Park SJ, Jung SH, Kim EJ, Jogeswar G, Ajita J (2012). miR-182 is a negative regulator of osteoblast proliferation, differentiation, and skeletogenesis through targeting FoxO1. J Bone Miner Res.

[CR112] Kim DY, Hwang I, Muller FL, Paik JH (2015). Functional regulation of FoxO1 in neural stem cell differentiation. Cell Death Differ.

[CR113] Kimura JO, Bolanos DM, Ricci L, Srivastava M (2022). Embryonic origins of adult pluripotent stem cells. Cell.

[CR114] Kitamura T, Kitamura YI, Funahashi Y, Shawber CJ, Castrillon DH, Kollipara R (2007). A Foxo/Notch pathway controls myogenic differentiation and fiber type specification. J Clin Invest.

[CR115] Klotz LO, Sanchez-Ramos C, Prieto-Arroyo I, Urbanek P, Steinbrenner H, Monsalve M (2015). Redox regulation of FoxO transcription factors. Redox Biol.

[CR116] Kobayashi Y, Furukawa-Hibi Y, Chen C, Horio Y, Isobe K, Ikeda K (2005). SIRT1 is critical regulator of FOXO-mediated transcription in response to oxidative stress. Int J Mol Med.

[CR117] Kojima K, Ohhashi R, Fujita Y, Hamada N, Akao Y, Nozawa Y (2008). A role for SIRT1 in cell growth and chemoresistance in prostate cancer PC3 and DU145 cells. Biochem Biophys Res Commun.

[CR118] Komander D (2009). The emerging complexity of protein ubiquitination. Biochem Soc Trans.

[CR119] Komori T (2010). Regulation of bone development and extracellular matrix protein genes by RUNX2. Cell Tissue Res.

[CR120] Komori T (2020). Functions of osteocalcin in bone, pancreas, testis, and muscle. Int J Mol Sci.

[CR121] Kops GJ, de Ruiter ND, De Vries-Smits AM, Powell DR, Bos JL, Burgering BM (1999). Direct control of the Forkhead transcription factor AFX by protein kinase B. Nature.

[CR122] Kuhn HG, Dickinson-Anson H, Gage FH (1996). Neurogenesis in the dentate gyrus of the adult rat: age-related decrease of neuronal progenitor proliferation. J Neurosci.

[CR123] Kuo M, Zilberfarb V, Gangneux N, Christeff N, Issad T (2008). O-glycosylation of FoxO1 increases its transcriptional activity towards the glucose 6-phosphatase gene. FEBS Lett.

[CR124] Kurakazu I, Akasaki Y, Hayashida M, Tsushima H, Goto N, Sueishi T (2019). FOXO1 transcription factor regulates chondrogenic differentiation through transforming growth factor beta1 signaling. J Biol Chem.

[CR125] Kuroda K, Tani S, Tamura K, Minoguchi S, Kurooka H, Honjo T (1999). Delta-induced Notch signaling mediated by RBP-J inhibits MyoD expression and myogenesis. J Biol Chem.

[CR126] Lamitina ST, Strange K (2005). Transcriptional targets of DAF-16 insulin signaling pathway protect C. elegans from extreme hypertonic stress. Am J Physiol Cell Physiol.

[CR127] Langlet F, Haeusler RA, Linden D, Ericson E, Norris T, Johansson A (2017). Selective inhibition of FOXO1 activator/repressor balance modulates hepatic glucose handling. Cell.

[CR128] Lee RY, Hench J, Ruvkun G (2001). Regulation of C. elegans DAF-16 and its human ortholog FKHRL1 by the daf-2 insulin-like signaling pathway. Curr Biol.

[CR129] Lee DY, Teyssier C, Strahl BD, Stallcup MR (2005). Role of protein methylation in regulation of transcription. Endocr Rev.

[CR130] Lee MJ, Yu GR, Yoo HJ, Kim JH, Yoon BI, Choi YK (2009). ANXA8 down-regulation by EGF-FOXO4 signaling is involved in cell scattering and tumor metastasis of cholangiocarcinoma. Gastroenterology.

[CR131] Lehtinen MK, Yuan Z, Boag PR, Yang Y, Villen J, Becker EB (2006). A conserved MST-FOXO signaling pathway mediates oxidative-stress responses and extends life span. Cell.

[CR132] Li L, Bhatia R (2011). Stem cell quiescence. Clin Cancer Res.

[CR133] Li Y, Guo W (2021). Neural stem cell niche and adult neurogenesis. Neuroscientist.

[CR134] Li F, Xie P, Fan Y, Zhang H, Zheng L, Gu D (2009). C terminus of Hsc70-interacting protein promotes smooth muscle cell proliferation and survival through ubiquitin-mediated degradation of FoxO1. J Biol Chem.

[CR135] Li Y, Yu J, Du D, Fu S, Chen Y, Yu F (2013). Involvement of post-transcriptional regulation of FOXO1 by HuR in 5-FU-induced apoptosis in breast cancer cells. Oncol Lett.

[CR136] Li D, Yuan Y, Wang D (2020). Regulation of response to nanopolystyrene by intestinal microRNA mir-35 in nematode Caenorhabditis elegans. Sci Total Environ.

[CR137] Li WJ, Wang CW, Tao L, Yan YH, Zhang MJ, Liu ZX (2021). Insulin signaling regulates longevity through protein phosphorylation in Caenorhabditis elegans. Nat Commun.

[CR138] Liang R, Ghaffari S (2017). Mitochondria and FOXO3 in stem cell homeostasis, a window into hematopoietic stem cell fate determination. J Bioenerg Biomembr.

[CR139] Liang XJ, Finkel T, Shen DW, Yin JJ, Aszalos A, Gottesman MM (2008). SIRT1 contributes in part to cisplatin resistance in cancer cells by altering mitochondrial metabolism. Mol Cancer Res.

[CR140] Liao L, Su X, Yang X, Hu C, Li B, Lv Y (2016). TNF-alpha inhibits FoxO1 by upregulating miR-705 to aggravate oxidative damage in bone marrow-derived mesenchymal stem cells during osteoporosis. Stem Cells.

[CR141] Libina N, Berman JR, Kenyon C (2003). Tissue-specific activities of C. elegans DAF-16 in the regulation of lifespan. Cell.

[CR142] Lin K, Dorman JB, Rodan A, Kenyon C (1997). daf-16: An HNF-3/forkhead family member that can function to double the life-span of Caenorhabditis elegans. Science.

[CR143] Link W (2019). Introduction to FOXO biology. Methods Mol Biol.

[CR144] Liu Z, Ambros V (1991). Alternative temporal control systems for hypodermal cell differentiation in Caenorhabditis elegans. Nature.

[CR145] Liu D, Yang X, Songyang Z (2000). Identification of CISK, a new member of the SGK kinase family that promotes IL-3-dependent survival. Curr Biol.

[CR146] Liu ZP, Wang Z, Yanagisawa H, Olson EN (2005). Phenotypic modulation of smooth muscle cells through interaction of Foxo4 and myocardin. Dev Cell.

[CR147] Liu K, Zhao Q, Liu P, Cao J, Gong J, Wang C (2016). ATG3-dependent autophagy mediates mitochondrial homeostasis in pluripotency acquirement and maintenance. Autophagy.

[CR148] Liu P, Liu K, Gu H, Wang W, Gong J, Zhu Y (2017). High autophagic flux guards ESC identity through coordinating autophagy machinery gene program by FOXO1. Cell Death Differ.

[CR149] Liu Y, Ao X, Ding W, Ponnusamy M, Wu W, Hao X (2018). Critical role of FOXO3a in carcinogenesis. Mol Cancer.

[CR150] Liu C, Li L, Yang B, Zhao Y, Dong X, Zhu L (2022). Transcriptome-wide N6-methyladenine methylation in granulosa cells of women with decreased ovarian reserve. BMC Genomics.

[CR151] Llorens-Bobadilla E, Zhao S, Baser A, Saiz-Castro G, Zwadlo K, Martin-Villalba A (2015). Single-cell transcriptomics reveals a population of dormant neural stem cells that become activated upon brain injury. Cell Stem Cell.

[CR152] Love DC, Ghosh S, Mondoux MA, Fukushige T, Wang P, Wilson MA (2010). Dynamic O-GlcNAc cycling at promoters of Caenorhabditis elegans genes regulating longevity, stress, and immunity. Proc Natl Acad Sci U S A.

[CR153] Lu C, Yang Z, Jiang S, Yang Y, Han Y, Lv J (2019). Forkhead box O4 transcription factor in human neoplasms: cannot afford to lose the novel suppressor. J Cell Physiol.

[CR154] Ludikhuize MC, Rodriguez Colman MJ (2021). Metabolic regulation of stem cells and differentiation: a Forkhead box O transcription factor perspective. Antioxid Redox Signal.

[CR155] Lynch VJ, Roth JJ, Wagner GP (2006). Adaptive evolution of Hox-gene homeodomains after cluster duplications. BMC Evol Biol.

[CR156] Magits W, Sablina AA (2022). The regulation of the protein interaction network by monoubiquitination. Curr Opin Struct Biol.

[CR157] Mansson R, Welinder E, Ahsberg J, Lin YC, Benner C, Glass CK (2012). Positive intergenic feedback circuitry, involving EBF1 and FOXO1, orchestrates B-cell fate. Proc Natl Acad Sci U S A.

[CR158] Marinkovic D, Zhang X, Yalcin S, Luciano JP, Brugnara C, Huber T (2007). Foxo3 is required for the regulation of oxidative stress in erythropoiesis. J Clin Invest.

[CR159] Martins R, Lithgow GJ, Link W (2016). Long live FOXO: unraveling the role of FOXO proteins in aging and longevity. Aging Cell.

[CR160] Matsuzaki H, Daitoku H, Hatta M, Tanaka K, Fukamizu A (2003). Insulin-induced phosphorylation of FKHR (Foxo1) targets to proteasomal degradation. Proc Natl Acad Sci U S A.

[CR161] Matsuzaki H, Daitoku H, Hatta M, Aoyama H, Yoshimochi K, Fukamizu A (2005). Acetylation of Foxo1 alters its DNA-binding ability and sensitivity to phosphorylation. Proc Natl Acad Sci U S A.

[CR162] Matsuzaki H, Ichino A, Hayashi T, Yamamoto T, Kikkawa U (2005). Regulation of intracellular localization and transcriptional activity of FOXO4 by protein kinase B through phosphorylation at the motif sites conserved among the FOXO family. J Biochem.

[CR163] McKinsey TA, Zhang CL, Olson EN (2001). Control of muscle development by dueling HATs and HDACs. Curr Opin Genet Dev.

[CR164] Mehta A, Zhao JL, Sinha N, Marinov GK, Mann M, Kowalczyk MS (2015). The microRNA-132 and microRNA-212 cluster regulates hematopoietic stem cell maintenance and survival with age by buffering FOXO3 expression. Immunity.

[CR165] Meyer KD, Jaffrey SR (2014). The dynamic epitranscriptome: N6-methyladenosine and gene expression control. Nat Rev Mol Cell Biol.

[CR166] Miyamoto K, Araki KY, Naka K, Arai F, Takubo K, Yamazaki S (2007). Foxo3a is essential for maintenance of the hematopoietic stem cell pool. Cell Stem Cell.

[CR167] Miyamoto K, Miyamoto T, Kato R, Yoshimura A, Motoyama N, Suda T (2008). FoxO3a regulates hematopoietic homeostasis through a negative feedback pathway in conditions of stress or aging. Blood.

[CR168] Morris BJ, Willcox DC, Donlon TA, Willcox BJ (2015). FOXO3: a major gene for human longevity–a mini-review. Gerontology.

[CR169] Motta MC, Divecha N, Lemieux M, Kamel C, Chen D, Gu W (2004). Mammalian SIRT1 represses forkhead transcription factors. Cell.

[CR170] Mouchiroud L, Houtkooper RH, Moullan N, Katsyuba E, Ryu D, Canto C (2013). The NAD(+)/Sirtuin pathway modulates longevity through activation of mitochondrial UPR and FOXO signaling. Cell.

[CR171] Mourikis P, Sambasivan R, Castel D, Rocheteau P, Bizzarro V, Tajbakhsh S (2012). A critical requirement for notch signaling in maintenance of the quiescent skeletal muscle stem cell state. Stem Cells.

[CR172] Munekata K, Sakamoto K (2009). Forkhead transcription factor Foxo1 is essential for adipocyte differentiation. In Vitro Cell Dev Biol Anim.

[CR173] Nagarajan A, Dogra SK, Sun L, Gandotra N, Ho T, Cai G (2017). Paraoxonase 2 facilitates pancreatic cancer growth and metastasis by stimulating GLUT1-mediated glucose transport. Mol Cell.

[CR174] Nakae J, Kitamura T, Kitamura Y, Biggs WH, Arden KC, Accili D (2003). The forkhead transcription factor Foxo1 regulates adipocyte differentiation. Dev Cell.

[CR175] Nasrin N, Ogg S, Cahill CM, Biggs W, Nui S, Dore J (2000). DAF-16 recruits the CREB-binding protein coactivator complex to the insulin-like growth factor binding protein 1 promoter in HepG2 cells. Proc Natl Acad Sci U S A.

[CR176] Natale F, Leone L, Rinaudo M, Sollazzo R, Barbati SA, La Greca F (2022). Neural stem cell-derived extracellular vesicles counteract insulin resistance-induced senescence of neurogenic niche. Stem Cells.

[CR177] Negishi Y, Ui N, Nakajima M, Kawashima K, Maruyama K, Takizawa T (2001). p21Cip-1/SDI-1/WAF-1 gene is involved in chondrogenic differentiation of ATDC5 cells in vitro. J Biol Chem.

[CR178] Neve A, Corrado A, Cantatore FP (2011). Osteoblast physiology in normal and pathological conditions. Cell Tissue Res.

[CR179] Nicaise AM, Willis CM, Crocker SJ, Pluchino S (2020). Stem cells of the aging brain. Front Aging Neurosci.

[CR180] Nowaczyk M, Malcher A, Zimna A, Rozwadowska N, Kurpisz M (2022). Effect of miR-195 inhibition on human skeletal muscle-derived stem/progenitor cells. Kardiol Pol.

[CR181] Obernier K, Alvarez-Buylla A (2019). Neural stem cells: origin, heterogeneity and regulation in the adult mammalian brain. Development.

[CR182] Obsil T, Obsilova V (2008). Structure/function relationships underlying regulation of FOXO transcription factors. Oncogene.

[CR183] Obsil T, Obsilova V (2011). Structural basis for DNA recognition by FOXO proteins. Biochim Biophys Acta.

[CR184] Ogg S, Paradis S, Gottlieb S, Patterson GI, Lee L, Tissenbaum HA (1997). The Fork head transcription factor DAF-16 transduces insulin-like metabolic and longevity signals in C. elegans. Nature.

[CR185] Oka K, Oka S, Sasaki T, Ito Y, Bringas P, Nonaka K (2007). The role of TGF-beta signaling in regulating chondrogenesis and osteogenesis during mandibular development. Dev Biol.

[CR186] Orea-Soufi A, Paik J, Braganca J, Donlon TA, Willcox BJ, Link W (2022). FOXO transcription factors as therapeutic targets in human diseases. Trends Pharmacol Sci.

[CR187] Orkin SH, Zon LI (2008). Hematopoiesis: an evolving paradigm for stem cell biology. Cell.

[CR188] Otsuki L, Brand AH (2020). Quiescent neural stem cells for brain repair and regeneration: lessons from model systems. Trends Neurosci.

[CR189] Owusu-Ansah E, Banerjee U (2009). Reactive oxygen species prime Drosophila haematopoietic progenitors for differentiation. Nature.

[CR190] Paik JH, Ding Z, Narurkar R, Ramkissoon S, Muller F, Kamoun WS (2009). FoxOs cooperatively regulate diverse pathways governing neural stem cell homeostasis. Cell Stem Cell.

[CR191] Papa L, Germain D (2014). SirT3 regulates the mitochondrial unfolded protein response. Mol Cell Biol.

[CR192] Park S, Lee Y, Pak JW, Kim H, Choi H, Kim JW (2015). O-GlcNAc modification is essential for the regulation of autophagy in Drosophila melanogaster. Cell Mol Life Sci.

[CR193] Pascual-Carreras E, Herrera-Ubeda C, Rossello M, Coronel-Cordoba P, Garcia-Fernandez J, Salo E (2021). Analysis of Fox genes in Schmidtea mediterranea reveals new families and a conserved role of Smed-foxO in controlling cell death. Sci Rep.

[CR194] Perrot V, Rechler MM (2005). The coactivator p300 directly acetylates the forkhead transcription factor Foxo1 and stimulates Foxo1-induced transcription. Mol Endocrinol.

[CR195] Pinho S, Frenette PS (2019). Haematopoietic stem cell activity and interactions with the niche. Nat Rev Mol Cell Biol.

[CR196] Pramanik KC, Fofaria NM, Gupta P, Srivastava SK (2014). CBP-mediated FOXO-1 acetylation inhibits pancreatic tumor growth by targeting SirT. Mol Cancer Ther.

[CR197] Puig O, Marr MT, Ruhf ML, Tjian R (2003). Control of cell number by Drosophila FOXO: downstream and feedback regulation of the insulin receptor pathway. Genes Dev.

[CR198] Qi W, Huang X, Neumann-Haefelin E, Schulze E, Baumeister R (2012). Cell-nonautonomous signaling of FOXO/DAF-16 to the stem cells of Caenorhabditis elegans. PLoS Genet.

[CR199] Qin Z, Hubbard EJ (2015). Non-autonomous DAF-16/FOXO activity antagonizes age-related loss of C. elegans germline stem/progenitor cells. Nat Commun.

[CR200] Rached MT, Kode A, Silva BC, Jung DY, Gray S, Ong H (2010). FoxO1 expression in osteoblasts regulates glucose homeostasis through regulation of osteocalcin in mice. J Clin Invest.

[CR201] Rached MT, Kode A, Xu L, Yoshikawa Y, Paik JH, Depinho RA (2010). FoxO1 is a positive regulator of bone formation by favoring protein synthesis and resistance to oxidative stress in osteoblasts. Cell Metab.

[CR202] Rahman MM, Stuchlick O, El-Karim EG, Stuart R, Kipreos ET, Wells L (2010). Intracellular protein glycosylation modulates insulin mediated lifespan in C. elegans. Aging (Albany NY).

[CR203] Rena G, Guo S, Cichy SC, Unterman TG, Cohen P (1999). Phosphorylation of the transcription factor forkhead family member FKHR by protein kinase B. J Biol Chem.

[CR204] Rena G, Prescott AR, Guo S, Cohen P, Unterman TG (2001). Roles of the forkhead in rhabdomyosarcoma (FKHR) phosphorylation sites in regulating 14-3-3 binding, transactivation and nuclear targetting. Biochem J.

[CR205] Rena G, Woods YL, Prescott AR, Peggie M, Unterman TG, Williams MR (2002). Two novel phosphorylation sites on FKHR that are critical for its nuclear exclusion. EMBO J.

[CR206] Renault VM, Rafalski VA, Morgan AA, Salih DA, Brett JO, Webb AE (2009). FoxO3 regulates neural stem cell homeostasis. Cell Stem Cell.

[CR207] Ro SH, Liu D, Yeo H, Paik JH (2013). FoxOs in neural stem cell fate decision. Arch Biochem Biophys.

[CR208] Rodda SJ, McMahon AP (2006). Distinct roles for Hedgehog and canonical Wnt signaling in specification, differentiation and maintenance of osteoblast progenitors. Development.

[CR209] Rodriguez-Colman MJ, Dansen TB, Burgering BMT (2023). FOXO transcription factors as mediators of stress adaptation. Nat Rev Mol Cell Biol.

[CR210] Rossi DJ, Bryder D, Zahn JM, Ahlenius H, Sonu R, Wagers AJ (2005). Cell intrinsic alterations underlie hematopoietic stem cell aging. Proc Natl Acad Sci U S A.

[CR211] Rossi DJ, Jamieson CH, Weissman IL (2008). Stems cells and the pathways to aging and cancer. Cell.

[CR212] Rossi L, Lin KK, Boles NC, Yang L, King KY, Jeong M (2012). Less is more: unveiling the functional core of hematopoietic stem cells through knockout mice. Cell Stem Cell.

[CR213] Salih DA, Rashid AJ, Colas D, de la Torre-Ubieta L, Zhu RP, Morgan AA (2012). FoxO6 regulates memory consolidation and synaptic function. Genes Dev.

[CR214] Sanphui P, Biswas SC (2013). FoxO3a is activated and executes neuron death via Bim in response to beta-amyloid. Cell Death Dis.

[CR215] Schaible R, Sussman M (2013). FOXO in aging: did evolutionary diversification of FOXO function distract it from prolonging life?. BioEssays.

[CR216] Schmitt-Ney M (2020). The FOXO’s advantages of being a family: considerations on function and evolution. Cells.

[CR217] Sekiya I, Tsuji K, Koopman P, Watanabe H, Yamada Y, Shinomiya K (2000). SOX9 enhances aggrecan gene promoter/enhancer activity and is up-regulated by retinoic acid in a cartilage-derived cell line, TC6. J Biol Chem.

[CR218] Selkoe DJ (2001). Alzheimer’s disease results from the cerebral accumulation and cytotoxicity of amyloid beta-protein. J Alzheimers Dis.

[CR219] Sharieh F, Eby JM, Roper PM, Callaci JJ (2020). Ethanol inhibits mesenchymal stem cell osteochondral lineage differentiation due in part to an activation of Forkhead box protein O-specific signaling. Alcohol Clin Exp Res.

[CR220] Sheikh MA, Emerald BS, Ansari SA (2021). Stem cell fate determination through protein O-GlcNAcylation. J Biol Chem.

[CR221] Shin H, Cha HJ, Na K, Lee MJ, Cho JY, Kim CY (2018). O-GlcNAcylation of the tumor suppressor FOXO3 triggers aberrant cancer cell growth. Cancer Res.

[CR222] Shiota M, Yokomizo A, Kashiwagi E, Tada Y, Inokuchi J, Tatsugami K (2010). Foxo3a expression and acetylation regulate cancer cell growth and sensitivity to cisplatin. Cancer Sci.

[CR223] Sigismund S, Polo S, Di Fiore PP (2004). Signaling through monoubiquitination. Curr Top Microbiol Immunol.

[CR224] Siqueira MF, Flowers S, Bhattacharya R, Faibish D, Behl Y, Kotton DN (2011). FOXO1 modulates osteoblast differentiation. Bone.

[CR225] Sousa-Victor P, Gutarra S, Garcia-Prat L, Rodriguez-Ubreva J, Ortet L, Ruiz-Bonilla V (2014). Geriatric muscle stem cells switch reversible quiescence into senescence. Nature.

[CR226] Srivastava M, Mazza-Curll KL, van Wolfswinkel JC, Reddien PW (2014). Whole-body acoel regeneration is controlled by Wnt and Bmp-Admp signaling. Curr Biol.

[CR227] Sudo K, Ema H, Morita Y, Nakauchi H (2000). Age-associated characteristics of murine hematopoietic stem cells. J Exp Med.

[CR228] Sun B, Li J, Shao D, Pan Y, Chen Y, Li S (2015). Adipose tissue-secreted miR-27a promotes liver cancer by targeting FOXO1 in obese individuals. Onco Targets Ther.

[CR229] Sun Y, Zhao J, Wu Q, Zhang Y, You Y, Jiang W (2022). Chondrogenic primed extracellular vesicles activate miR-455/SOX11/FOXO axis for cartilage regeneration and osteoarthritis treatment. NPJ Regen Med.

[CR230] Takahashi Y, Daitoku H, Hirota K, Tamiya H, Yokoyama A, Kako K (2011). Asymmetric arginine dimethylation determines life span in C. elegans by regulating forkhead transcription factor DAF-16. Cell Metab.

[CR231] Takaishi H, Konishi H, Matsuzaki H, Ono Y, Shirai Y, Saito N (1999). Regulation of nuclear translocation of forkhead transcription factor AFX by protein kinase B. Proc Natl Acad Sci U S A.

[CR232] Teixeira CC, Liu Y, Thant LM, Pang J, Palmer G, Alikhani M (2010). Foxo1, a novel regulator of osteoblast differentiation and skeletogenesis. J Biol Chem.

[CR233] Thakurela S, Tiwari N, Schick S, Garding A, Ivanek R, Berninger B (2016). Mapping gene regulatory circuitry of Pax6 during neurogenesis. Cell Discov.

[CR234] Tissenbaum HA, Guarente L (2001). Increased dosage of a sir-2 gene extends lifespan in Caenorhabditis elegans. Nature.

[CR235] Tothova Z, Gilliland DG (2007). FoxO transcription factors and stem cell homeostasis: insights from the hematopoietic system. Cell Stem Cell.

[CR236] Tothova Z, Kollipara R, Huntly BJ, Lee BH, Castrillon DH, Cullen DE (2007). FoxOs are critical mediators of hematopoietic stem cell resistance to physiologic oxidative stress. Cell.

[CR237] Tsai KL, Sun YJ, Huang CY, Yang JY, Hung MC, Hsiao CD (2007). Crystal structure of the human FOXO3a-DBD/DNA complex suggests the effects of post-translational modification. Nucleic Acids Res.

[CR238] Tsai WB, Chung YM, Takahashi Y, Xu Z, Hu MC (2008). Functional interaction between FOXO3a and ATM regulates DNA damage response. Nat Cell Biol.

[CR239] Tureckova J, Wilson EM, Cappalonga JL, Rotwein P (2001). Insulin-like growth factor-mediated muscle differentiation: collaboration between phosphatidylinositol 3-kinase-Akt-signaling pathways and myogenin. J Biol Chem.

[CR240] Urbanek P, Klotz LO (2017). Posttranscriptional regulation of FOXO expression: microRNAs and beyond. Br J Pharmacol.

[CR241] Van Der Heide LP, Hoekman MF, Smidt MP (2004). The ins and outs of FoxO shuttling: mechanisms of FoxO translocation and transcriptional regulation. Biochem J.

[CR242] van der Heide LP, Smidt MP (2005). Regulation of FoxO activity by CBP/p300-mediated acetylation. Trends Biochem Sci.

[CR243] van der Heide LP, Jacobs FM, Burbach JP, Hoekman MF, Smidt MP (2005). FoxO6 transcriptional activity is regulated by Thr26 and Ser184, independent of nucleo-cytoplasmic shuttling. Biochem J.

[CR244] van der Horst A, de Vries-Smits AM, Brenkman AB, van Triest MH, van den Broek N, Colland F (2006). FOXO4 transcriptional activity is regulated by monoubiquitination and USP7/HAUSP. Nat Cell Biol.

[CR245] Vilchez D, Boyer L, Morantte I, Lutz M, Merkwirth C, Joyce D (2012). Increased proteasome activity in human embryonic stem cells is regulated by PSMD11. Nature.

[CR246] Vilchez D, Boyer L, Lutz M, Merkwirth C, Morantte I, Tse C (2013). FOXO4 is necessary for neural differentiation of human embryonic stem cells. Aging Cell.

[CR247] von Groote-Bidlingmaier F, Schmoll D, Orth HM, Joost HG, Becker W, Barthel A (2003). DYRK1 is a co-activator of FKHR (FOXO1a)-dependent glucose-6-phosphatase gene expression. Biochem Biophys Res Commun.

[CR248] Wang MC, Bohmann D, Jasper H (2005). JNK extends life span and limits growth by antagonizing cellular and organism-wide responses to insulin signaling. Cell.

[CR249] Wang M, Zhang X, Zhao H, Wang Q, Pan Y (2009). FoxO gene family evolution in vertebrates. BMC Evol Biol.

[CR250] Wang F, Chan CH, Chen K, Guan X, Lin HK, Tong Q (2012). Deacetylation of FOXO3 by SIRT1 or SIRT2 leads to Skp2-mediated FOXO3 ubiquitination and degradation. Oncogene.

[CR251] Wang F, Marshall CB, Yamamoto K, Li GY, Gasmi-Seabrook GM, Okada H (2012). Structures of KIX domain of CBP in complex with two FOXO3a transactivation domains reveal promiscuity and plasticity in coactivator recruitment. Proc Natl Acad Sci U S A.

[CR252] Wang F, Marshall CB, Ikura M (2015). Forkhead followed by disordered tail: the intrinsically disordered regions of FOXO3a. Intrinsically Disord Proteins.

[CR253] Wang C, Li Y, Zeng L, Shi C, Peng Y, Li H (2022). Tris(1,3-dichloro-2-propyl) phosphate reduces longevity through a specific microRNA-mediated DAF-16/FoxO in an unconventional insulin/insulin-like growth factor-1 signaling pathway. J Hazard Mater.

[CR254] Warr MR, Binnewies M, Flach J, Reynaud D, Garg T, Malhotra R (2013). FOXO3A directs a protective autophagy program in haematopoietic stem cells. Nature.

[CR255] Webb AE, Brunet A (2014). FOXO transcription factors: key regulators of cellular quality control. Trends Biochem Sci.

[CR256] Webb AE, Pollina EA, Vierbuchen T, Urban N, Ucar D, Leeman DS (2013). FOXO3 shares common targets with ASCL1 genome-wide and inhibits ASCL1-dependent neurogenesis. Cell Rep.

[CR257] Weigel D, Jurgens G, Kuttner F, Seifert E, Jackle H (1989). The homeotic gene fork head encodes a nuclear protein and is expressed in the terminal regions of the Drosophila embryo. Cell.

[CR258] Weigelt J, Climent I, Dahlman-Wright K, Wikstrom M (2001). Solution structure of the DNA binding domain of the human forkhead transcription factor AFX (FOXO4). Biochemistry.

[CR259] Wen Y, Bi P, Liu W, Asakura A, Keller C, Kuang S (2012). Constitutive Notch activation upregulates Pax7 and promotes the self-renewal of skeletal muscle satellite cells. Mol Cell Biol.

[CR260] Weng Q, Liu Z, Li B, Liu K, Wu W, Liu H (2016). Oxidative stress induces mouse follicular granulosa cells apoptosis via JNK/FoxO1 pathway. PLoS One.

[CR261] Wilson A, Trumpp A (2006). Bone-marrow haematopoietic-stem-cell niches. Nat Rev Immunol.

[CR262] Wirick MJ, Cale AR, Smith IT, Alessi AF, Starostik MR, Cuko L (2021). daf-16/FOXO blocks adult cell fate in Caenorhabditis elegans dauer larvae via lin-41/TRIM71. PLoS Genet.

[CR263] Woods YL, Rena G, Morrice N, Barthel A, Becker W, Guo S (2001). The kinase DYRK1A phosphorylates the transcription factor FKHR at Ser329 in vitro, a novel in vivo phosphorylation site. Biochem J.

[CR264] Wu AL, Kim JH, Zhang C, Unterman TG, Chen J (2008). Forkhead box protein O1 negatively regulates skeletal myocyte differentiation through degradation of mammalian target of rapamycin pathway components. Endocrinology.

[CR265] Xie Q, Hao Y, Tao L, Peng S, Rao C, Chen H (2012). Lysine methylation of FOXO3 regulates oxidative stress-induced neuronal cell death. EMBO Rep.

[CR266] Xu X, Leng J, Zhang X, Capellini TD, Chen Y, Yang L (2021). Identification of IGF2BP1-related lncRNA-miRNA-mRNA network in goat skeletal muscle satellite cells. Anim Sci J.

[CR267] Yalcin S, Zhang X, Luciano JP, Mungamuri SK, Marinkovic D, Vercherat C (2008). Foxo3 is essential for the regulation of ataxia telangiectasia mutated and oxidative stress-mediated homeostasis of hematopoietic stem cells. J Biol Chem.

[CR268] Yamagata K, Daitoku H, Takahashi Y, Namiki K, Hisatake K, Kako K (2008). Arginine methylation of FOXO transcription factors inhibits their phosphorylation by Akt. Mol Cell.

[CR269] Yan H, Li Q, Wu J, Hu W, Jiang J, Shi L (2017). MiR-629 promotes human pancreatic cancer progression by targeting FOXO3. Cell Death Dis.

[CR270] Yang Y, Hou H, Haller EM, Nicosia SV, Bai W (2005). Suppression of FOXO1 activity by FHL2 through SIRT1-mediated deacetylation. EMBO J.

[CR271] Yang JY, Zong CS, Xia W, Yamaguchi H, Ding Q, Xie X (2008). ERK promotes tumorigenesis by inhibiting FOXO3a via MDM2-mediated degradation. Nat Cell Biol.

[CR272] Yang S, Xu H, Yu S, Cao H, Fan J, Ge C (2011). Foxo1 mediates insulin-like growth factor 1 (IGF1)/insulin regulation of osteocalcin expression by antagonizing Runx2 in osteoblasts. J Biol Chem.

[CR273] Yeo H, Lyssiotis CA, Zhang Y, Ying H, Asara JM, Cantley LC (2013). FoxO3 coordinates metabolic pathways to maintain redox balance in neural stem cells. EMBO J.

[CR274] Yin H, Price F, Rudnicki MA (2013). Satellite cells and the muscle stem cell niche. Physiol Rev.

[CR275] You H, Yamamoto K, Mak TW (2006). Regulation of transactivation-independent proapoptotic activity of p53 by FOXO3a. Proc Natl Acad Sci U S A.

[CR276] Yu F, Jin L, Yang G, Ji L, Wang F, Lu Z (2014). Post-transcriptional repression of FOXO1 by QKI results in low levels of FOXO1 expression in breast cancer cells. Oncol Rep.

[CR277] Yuan Z, Becker EB, Merlo P, Yamada T, DiBacco S, Konishi Y (2008). Activation of FOXO1 by Cdk1 in cycling cells and postmitotic neurons. Science.

[CR278] Yuan Z, Lehtinen MK, Merlo P, Villen J, Gygi S, Bonni A (2009). Regulation of neuronal cell death by MST1-FOXO1 signaling. J Biol Chem.

[CR279] Yuan S, Zhang L, Ji L, Zhong S, Jiang L, Wan Y (2022). FoxO3a cooperates with RUNX1 to promote chondrogenesis and terminal hypertrophic of the chondrogenic progenitor cells. Biochem Biophys Res Commun.

[CR280] Zhang H, Wang ZZ (2008). Mechanisms that mediate stem cell self-renewal and differentiation. J Cell Biochem.

[CR281] Zhang X, Gan L, Pan H, Guo S, He X, Olson ST (2002). Phosphorylation of serine 256 suppresses transactivation by FKHR (FOXO1) by multiple mechanisms. Direct and indirect effects on nuclear/cytoplasmic shuttling and DNA binding. J Biol Chem.

[CR282] Zhang B, Tomita Y, Ch’ng E, Qiu Y, He J, Jin YF (2009). Prognostic significance of phosphorylated FOXO1 expression in soft tissue sarcoma. Ann Surg Oncol.

[CR283] Zhang H, Pan Y, Zheng L, Choe C, Lindgren B, Jensen ED (2011). FOXO1 inhibits Runx2 transcriptional activity and prostate cancer cell migration and invasion. Cancer Res.

[CR284] Zhang X, Yalcin S, Lee DF, Yeh TY, Lee SM, Su J (2011). FOXO1 is an essential regulator of pluripotency in human embryonic stem cells. Nat Cell Biol.

[CR285] Zhang L, Cai M, Gong Z, Zhang B, Li Y, Guan L (2017). Geminin facilitates FoxO3 deacetylation to promote breast cancer cell metastasis. J Clin Invest.

[CR286] Zhang XS, Wang T, Lin XW, Denlinger DL, Xu WH (2017). Reactive oxygen species extend insect life span using components of the insulin-signaling pathway. Proc Natl Acad Sci U S A.

[CR287] Zhang LY, Chen Y, Jia J, Zhu X, He Y, Wu LM (2019). MiR-27a promotes EMT in ovarian cancer through active Wnt/. Cancer Biomark.

[CR288] Zhao HH, Herrera RE, Coronado-Heinsohn E, Yang MC, Ludes-Meyers JH, Seybold-Tilson KJ (2001). Forkhead homologue in rhabdomyosarcoma functions as a bifunctional nuclear receptor-interacting protein with both coactivator and corepressor functions. J Biol Chem.

[CR289] Zhao Y, Yang J, Liao W, Liu X, Zhang H, Wang S (2010). Cytosolic FoxO1 is essential for the induction of autophagy and tumour suppressor activity. Nat Cell Biol.

